# Biomimetic Gradient Lubrication Hydrogel Contrived by Self-Reinforced MOFs Nanoparticle Network

**DOI:** 10.1007/s40820-025-02001-x

**Published:** 2026-01-12

**Authors:** Desheng Liu, Yixian Wang, Changcheng Bai, Danli Hu, Xingxing Yang, Yaozhong Lu, Tao Wu, Fei Zhai, Pan Jiang, Xiaolong Wang, Weimin Liu

**Affiliations:** 1 State Key Laboratory of Solid Lubrication, Lanzhou Institute of Chemical Physics, Chinese Academy of Sciences, Lanzhou, 730000 People’s Republic of China; 2 School of Chemistry and Chemical Engineering, State Key Laboratory Incubation Base for Green, Processing of Chemical Engineering, Shihezi University, Shihezi, 832003 People’s Republic of China; 3 Shandong Laboratory of Advanced Materials and Green Manufacturing at Yantai, Yantai Zhongke Research Institute of Advanced Materials and Green Chemical Engineering, Yantai, 264006 People’s Republic of China

**Keywords:** Biomimetic gradient architecture, DIW 3D printing, Lubricating hydrogel, MOFs nanoparticle network, Slippery meniscus

## Abstract

The development of gradient lubrication materials is critical for numerous biomedical applications, particularly in magnifying mechanical properties and service longevity. Herein, we present an innovative approach to fabricate biomimetic gradient lubrication hydrogel through the synergistic integration of three-dimensional (3D) printed metal–organic frameworks (MOFs) nanoparticle network hydrogel skeletons with bio-inspired lubrication design. Specifically, robust hydrogel skeletons were engineered through single or multi-material 3D printing, followed by the in situ growth of MOFs nanoparticles within this hydrogel network to create a reinforced, load-bearing architecture. Subsequently, biomimetic lubrication capability was enabled by mechanically coupling another lubricating hydrogel within 3D-printed MOFs nanoparticle network hydrogel skeleton. The superficial layer is highly lubricious to ensure low coefficient of friction (~ 0.1141) and wear resistance (40,000 cycles), while the deeper layer is stiffer to afford the obligatory mechanical support (fracture strength ~ 2.50 MPa). Furthermore, the gradient architecture stiffness of the hydrogel can be modulated by manipulating the spatial distribution of MOFs within the 3D-printed hydrogel skeleton. As a proof-of-concept, biomimetic gradient hydrogel meniscus structures with C- and O-shaped configurations were constructed by leveraging multi-material 3D printing, demonstrating exceptional lubrication performance. This innovative biomimetic design opens new avenues for creating implantable biomedical gradient lubricating materials with reinforced mechanical and lubrication performance.

**Supplementary Information:**

The online version contains supplementary material available at 10.1007/s40820-025-02001-x.

## Highlights

- Self-reinforced network of metal-organic frameworks nanoparticles significantly improved the mechanical strength and durability of the hydrogel.
- Biomimetic lubricating hydrogels with architectural and compositional gradients enabled by multi-material 3D printing.
- Slippery hydrogel meniscus substitutes with complicated gradient structures and reliable cushioning layers were manufactured.

**Supplementary Information:**

The online version contains supplementary material available at 10.1007/s40820-025-02001-x.

## Graphical abstract

**Figure:**
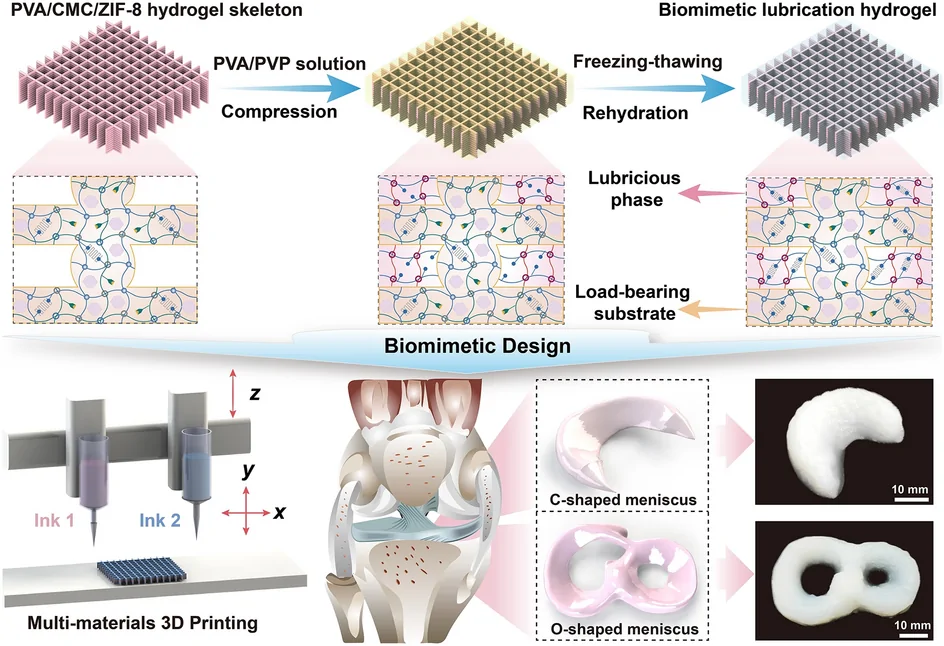

## Introduction

Hydrogels, composed of water-filled, three-dimensional cross-linked polymer chains, have been extensively investigated for their unique properties, such as high water content, tunable mechanics, and favorable biocompatibility [1–3]. Moreover, hydrogel’s attributes like soft, wet, and slippery resemble biological tissues, making them an ideal candidate for multitudinous biomedical applications [4–6], particularly in tissue replacements and artificial organs [7, 8]. Despite these benefits, however, conventional hydrogels often suffer from drawbacks in mechanical strength, wear resistance, and durable lubrication, which restricts their usability in many applications, particularly those involving repetitive, dynamic, and long-term load-bearing circumstances. To overcome these limitations, several approaches have been developed to ameliorate the performance of hydrogels, such as microphase separation [9, 10], hydrogen bond [11, 12], crystalline domain [13–15], and reinforcement with nanomaterials [16]. Surprisingly, the incorporation of nanomaterials or nanoparticles into the hydrogel network will exceedingly strengthen their mechanical properties. Among the numerous hydrogel’s nanoparticle reinforcements, the integration of MOFs nanoparticles into hydrogels enables the establishment of self-reinforcing networks, which is considerably beneficial in strengthening the overall mechanical strength and load-bearing capacity [17–19]. Furthermore, the MOFs network within the hydrogel is more resistant to deformation under mechanical stress, which is conductive to mitigate wear and reduce friction [20, 21]. Therefore, the self-reinforcing nature of the MOFs nanoparticles is a critical factor in the durability and longevity of the hydrogel. Unlike much prior research that primarily exploit MOFs nanoparticles as reinforcing fillers [22, 23], here the MOFs nanoparticle network serves as a multifunctional cross-linking point within the hydrogel matrix that enables the significant enhancement of the mechanical performance of engineered hydrogels. To date, the fabrication of MOFs nanoparticle-reinforced hydrogels can be achieved through several manners, including in situ growth of MOFs within the hydrogel matrix, or by embedding pre-synthesized MOFs into the hydrogel network [24–26]. While the integration of MOFs within the hydrogel network is able to reinforce mechanical performance, the ability to fabricate self-reinforcing MOFs nanocomposite hydrogels with precise control over macro- and micro-structures, such as sophisticated gradients, remains a serious challenge, particularly, because traditional homogeneous hydrogel preparation approaches are inherently unable to generate such gradients.

Despite advances in MOFs nanocomposite hydrogels, conventional bulk or homogeneous ones lack the spatially organized complexity of natural tissues like cartilage. To this end, the incorporation of biomimetic gradient principles into hydrogel design aims to enhance mechanical performance, avoid stress concentrations, and enable multifunctionality within a single construct [27–29]. Captivatingly, direct-ink-writing (DIW) 3D printing techniques have opened new avenues for fabricating complex hydrogel structures that replicate the mechanical gradient of biological tissues by leveraging unparalleled spatial control [30–32]. Nevertheless, standard DIW 3D printing often only extrudes one type of ink, making it difficult to achieve mechanical gradient hydrogel structures. Multi-nozzles DIW with switchable inks enable the stacking of different materials or components in a controlled manner, allowing the fabrication of biomimetic gradient hydrogels with variable mechanical properties throughout the structure [30, 33–35]. The similar shear-thinning behaviors of the hydrogels, despite their distinct mechanical properties, enable precise co-extrude and strong interfacial bonding during DIW, thus creating robust hydrogel gradient architectures with tailored mechanical properties. Thereupon, the creation of biomimetic structured hydrogels with compositions gradients, structural gradients, and mechanical gradients through DIW 3D printing is a critical innovation. For example, DIW 3D printing can be utilized to create hydrogels with spatially varying stiffness, which mimics the properties of biological tissues with different mechanical requirements across the structure, such as cartilage and meniscus [36–38]. Furthermore, DIW 3D-printed biomimetic gradient hydrogels can tailor lubrication behavior to specific regions of the hydrogel based on mechanical stress [39, 40]. To this end, we hypothesize that the gradient design enables the creation of hydrogels with improved mechanical performance, better wear resistance, and enhanced persistent lubrication. Despite the potential advantages, several challenges remain in the development of biomimetic hydrogels with graded mechanical and lubrication properties. One promising approach involves the use of gradient structures and MOFs nanoparticles reinforcement to improve the performance of hydrogels in high-stress environments. Consequently, the development of 3D-printed biomimetic gradient lubrication hydrogels reinforced with MOFs nanoparticle networks represents a cutting-edge advancement, making them ideal candidates for applications that require friction reduction and wear prevention, such as meniscus replacements.

Herein, we proposed a pioneering approach to fabricate biomimetic gradient lubrication hydrogel by leveraging the synergistic strategies of MOFs nanoparticle self-reinforcing network, DIW 3D printing technique, and interfacial mechanical interlocking. Specifically, a robust load-bearing skeleton was engineered through DIW 3D printing of PVA/CMC hydrogel containing MOFs precursor and was then subjected to in situ growth to form a self-reinforced MOFs nanoparticle network. To achieve biomimetic gradient lubrication surface that mimics the natural lubrication mechanisms, lubricious PVA/PVP hydrogel was incorporated into the 3D-printed load-bearing skeleton through interfacial mechanical interlocking. This creates a persistent lubrication layer on the surface of the MOFs nanoparticles network hydrogel, which is critical for long-term wear resistance, while also addressing a common failure issue of hydrogel lubricity diminishing over time. Furthermore, the gradient design enabled by harnessing multi-material 3D printing allows for precise spatial distribution of MOFs nanoparticles and tailored lubrication properties. Meanwhile, several hydrogel meniscus surrogates with gradient structures and superior lubricity are also capable of fabricating by leveraging multi-material 3D printing. Thereupon, by mimicking natural lubrication mechanisms and harnessing the unparalleled self-reinforcing properties of MOFs network, this customizable biomimetic gradient hydrogel offers a versatile solution for a wide range of specific application scenarios.

## Results and Discussion

### Design and Fabrication of Biomimetic Gradient Lubrication Hydrogel with MOFs Nanoparticle Self-Reinforced Network

**Fig. 1:**
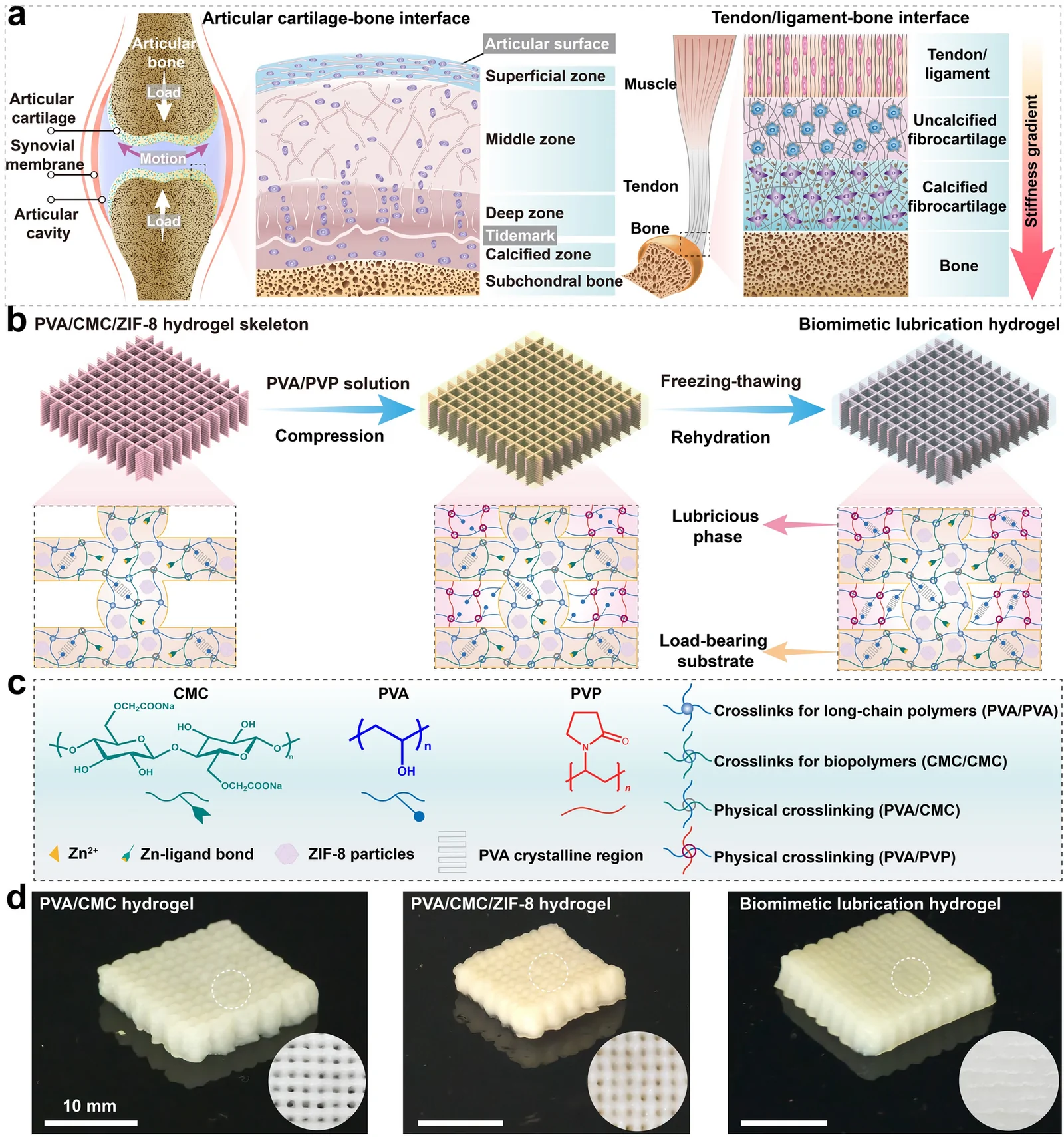
Design and fabrication of biomimetic gradient lubrication hydrogel reinforced by harnessing 3D-printed MOFs nanoparticle network. **a** Schematic depiction of the gradient structure of the soft–hard tissue interfaces, including the articular cartilage–bone and tendon/ligament–bone interfaces. **b** Schematic illustration of the fabrication of biomimetic gradient lubrication hydrogel composed of MOFs nanoparticle network hydrogel load-bearing phase and PVA/PVP hydrogel lubricating phase. **c** Diverse components for the preparation of biomimetic gradient lubricated hydrogels and the multiple interactions present in hydrogel network. **d** Photographs of engineered PVA/CMC, PVA/CMC/ZIF-8, and biomimetic gradient lubrication hydrogels

In nature, the soft–hard interfaces of numerous biological tissues showcase gradients in their structure and function [41, 42], spanning the cartilage–bone interface, tendon–bone interface, ligament–bone interface, and so on (Fig. 1a). A classic example is the cartilage-to-bone interface, which is composed of superficial, transitional, deep, calcified cartilage, and subchondral zones, whose mechanical properties vary from the surface to the deeper layers, enabling the tissue to absorb shock, reduce friction, and sustain load-bearing [43]. Furthermore, in the tendon or ligament-to-bone interface, there are four individual gradient regions, including bone, mineralized fibrocartilage, unmineralized fibrocartilage, and ligament/tendon tissues [44]. Thereupon, these interfaces consist of complex gradient structures in mechanical and biochemical properties, conferring them high stiffness and impact resistance.

To enable this mechanical gradient, we propose a novel biomimetic gradient lubrication hydrogel reinforced by MOFs nanoparticle network in combination with a biomimetic gradient lubrication approach and DIW 3D printing technique. To fabricate a robust hydrogel load-bearing skeleton with tunable mechanical properties, 12.5 wt% polyvinyl alcohol (PVA) sol, sodium carboxymethyl cellulose (CMC), and 2-methylimidazole (2-MIM) were homogeneously blended to obtain precursor inks, and then, PVA/CMC hydrogels with PVA crystalline domains were created by DIW 3D printing and freezing–thawing cycles treatment (Fig. S1). Subsequently, PVA/CMC hydrogel undergone in situ growth of ZIF-8 nanoparticles, metal coordination cross-linking, and rehydration process, transforming them into PVA/CMC/ZIF-8 hydrogel with MOFs nanoparticle self-reinforcing network. Furthermore, 3D-printed PVA/CMC/ZIF-8 hydrogel manifests various physical cross-linking interactions, including multiple H-bonding networks (e.g., between CMCs, between PVAs, as well as between CMC and PVA), PVA crystallization network, MOFs nanoparticle network, and metal coordination network, which is conducive to modulating and strengthening their mechanical properties.

To construct biomimetic gradient lubrication hydrogel composed of load-bearing phase and lubricating phase, furthermore, the 3D-printed MOFs nanoparticle network hydrogel was served as the load-bearing skeleton, and then, lubricious PVA/PVP hydrogel was integrated into stereoplasm skeletons through interfacial mechanical interlocking (Fig. 1b). Additionally, there are miscellaneous interactions in the biomimetic gradient lubricated hydrogels enhanced by MOFs nanoparticle networks, including PVA crystalline domain, metal coordination, and multiple H-bonding cross-linking networks (Fig. 1c). The prepared PVA/CMC, PVA/CMC/ZIF-8, and biomimetic gradient hydrogels are disclosed in Fig. 1d, where the biomimetic gradient lubricated hydrogels have a slippery surface. This innovative biomimetic approach and multiple interactions will be propitious to meliorate the interfacial toughness and binding strength of biomimetic gradient hydrogels, which in turn are conducive to actualizing the superior load-bearing capacities and lubrication properties across varying stress and motion conditions.

### Evaluation on Extrudability and Printability of Self-Reinforcing MOFs Nanoparticle Network Hydrogel Inks

**Fig. 2:**
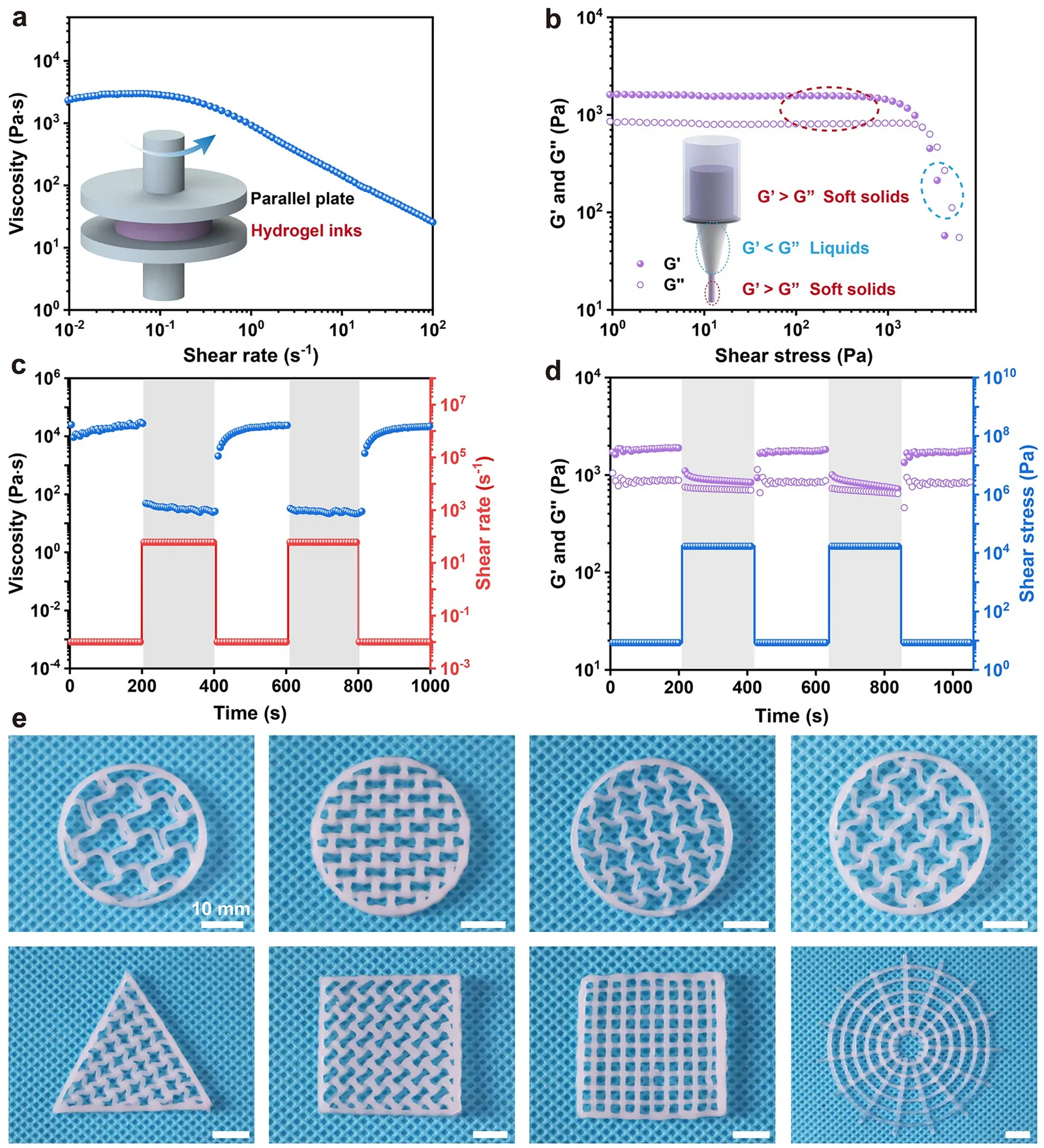
Extrudability and printability assessments of self-reinforcing MOFs nanoparticle network hydrogel inks. **a** Shear-thinning behavior of hydrogel ink. **b** G' and G'' of hydrogel ink. **c** Viscosity thixotropic restoration of hydrogel ink. **d** Variation in G′ and G" of hydrogel ink under high and low alternating stress. **e** Various free-standing MOFs nanoparticle network hydrogel structures with different filling patterns and dimensions

For DIW 3D-printed hydrogel architectures, printability is closely associated with multiple rheological parameters of the hydrogel ink and ordinarily referring to extrudability, filament regularity, and shape fidelity. To this end, the non-Newtonian behavior of self-reinforcing MOFs nanoparticle network hydrogel ink was investigated by rheological examination at shear rates ranging from 0.01 to 100 s^−1^, as illustrated in Fig. 2a. As shown in Fig. 2a, the viscosity of hydrogel ink steadily declines with the increase of shear rate, indicating that exemplary shear-thinning characteristics are conducive to the production of uninterrupted filamentous morphology. As shown in Fig. 2b, the strain amplitude sweeps further showcased that the hydrogel ink displays a high yield stress (~ 2.05 kPa). Furthermore, the storage modulus (G') of the ink during the shearing process is higher than the loss modulus (G''), which illustrates solid-like characteristics and is beneficial to impeding the collapse and distortion of the architecture during the layer-by-layer stacking manufacturing process. Henceforth, alternating sweeps of hydrogel ink at high (60 s^−1^) and low (0.01 s^−1^) shear rates showed superior shear-thinning behavior and quick thixotropic recovery characteristics, ensuring the creation of high shape fidelity structures (Fig. 2c). Based on the variation of G' and G" of the hydrogel ink at the alternating sweep of low (10 Pa) and high (16,800 Pa) shear stresses, it was found that the ink could meet the requirements of extrusion printing to achieve high geometrical precision and structural integrity of 3D-printed multilayered structures (Fig. 2d). To exemplify extrudability, printability, and shape fidelity of hydrogel ink, several proof-of-concept MOFs nanoparticle network self-strengthening hydrogel architectures with different shapes, dimensions, and patterns were constructed by DIW 3D printing with a conical nozzle of a diameter of 510 μm, as revealed in Fig. 2e. These 3D-printed MOFs nanoparticle network hydrogel structures exhibit uniform grids and interconnectivity. Moreover, the hydrogel filaments between adjacent layers were well stacked without collapsing due to the well self-supporting of the ink, underscoring that the obtained 3D-printed hydrogel structures own high shape fidelity and structural integrity. Thereupon, these results further manifest that the self-reinforcing MOFs nanoparticle network hydrogel ink retains acceptable stackability throughout the DIW 3D printing process, ensuring better fidelity of the final 3D-printed hydrogel structures.

### Structural Evolution and Characterization of Self-Reinforced MOFs Nanoparticle Network Hydrogel

**Fig. 3:**
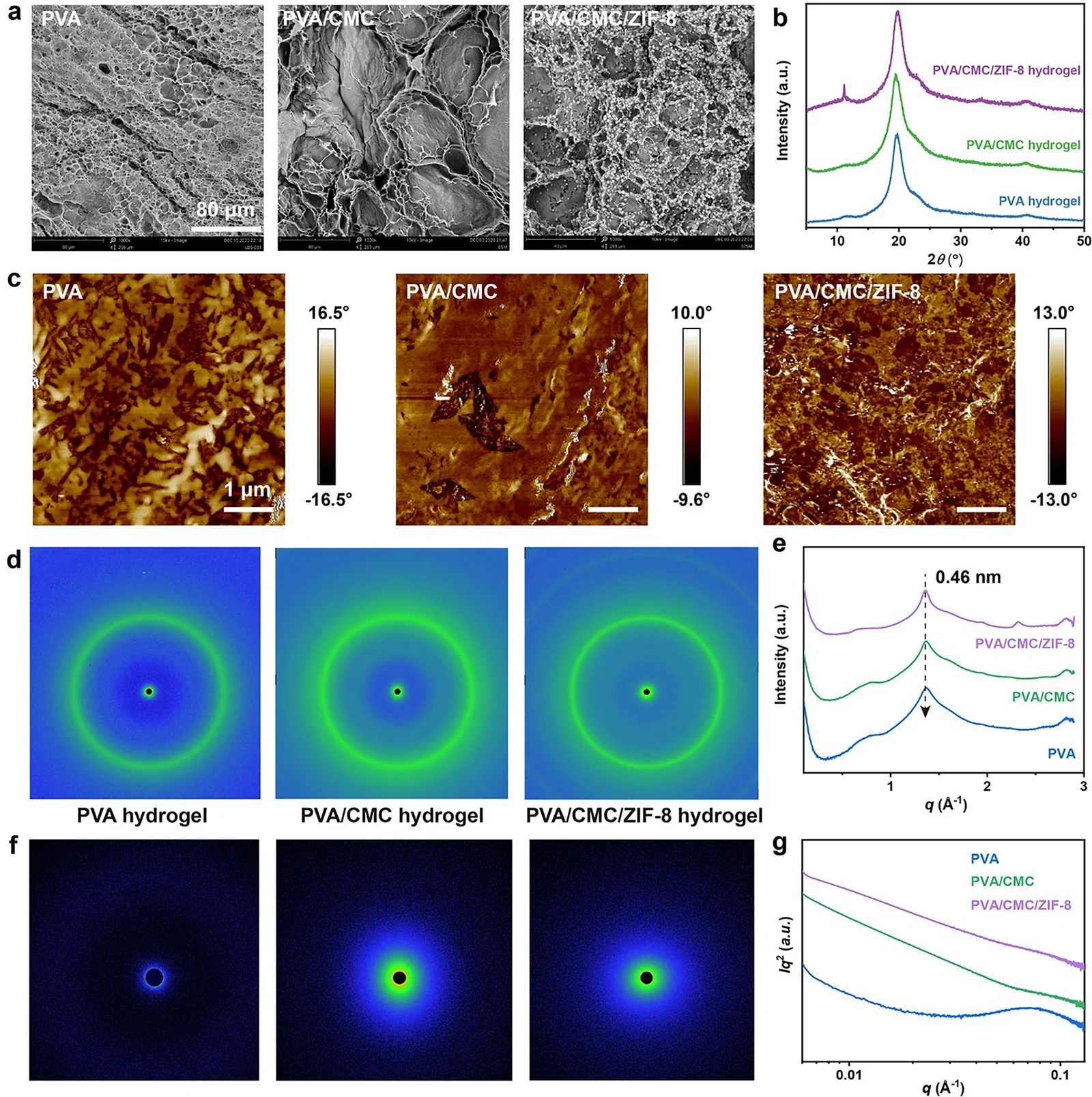
Structural evolution and characterization of self-reinforced MOFs nanoparticle network hydrogel. **a** SEM images, **b** XRD spectra, and **c** AFM phase diagrams of the PVA, PVA/CMC, and PVA/CMC/ZIF-8 hydrogels. **d** 2D WAXS patterns and corresponding **e** 1D WAXS curves of the PVA, PVA/CMC, and PVA/CMC/ZIF-8 hydrogels. **f** 2D SAXS patterns and corresponding g 1D SAXS profiles of the PVA, PVA/CMC, and PVA/CMC/ZIF-8 hydrogels

As revealed in Fig. 3a, the microscopic morphological and structural transmutation of PVA, PVA/CMC, and PVA/CMC/ZIF-8 hydrogels was distinguished by scanning electron microscopy (SEM). Compared with PVA hydrogel, the network structure of PVA/CMC hydrogels is dense due to the strong H-bonding interaction between PVA and CMC. For the PVA/CMC/ZIF-8 hydrogel, in contrast, there are numerous ZIF-8 nanoparticles in the network structure. It is worth mentioning that the wettability of the PVA/CMC/ZIF-8 hydrogel with MOFs nanoparticle network changes greatly (Fig. S2). Subsequently, X-ray diffraction (XRD) was leveraged to validate the crystallinity of PVA, PVA/CMC, and PVA/CMC/ZIF-8 hydrogels. Compared with PVA and PVA/CMC hydrogels, as shown in Fig. 3b, the diffraction peak of PVA/CMC/ZIF-8 hydrogel at 2*θ* = 11.2° indicates the formation of ZIF-8 nanoparticles in the hydrogel network [45]. Meanwhile, all hydrogels have a sharp diffraction peak at 2*θ* = 19.7°, which corresponds to the (101) diffraction plane of the semi-crystalline PVA [46]. The differential scanning calorimetry (DSC) curves have a distinct endothermic peak from 200 to 240 °C, which is the molten crystallization peak of PVA (Fig. S3) [47]. The characteristic peaks of PVA/CMC/ZIF-8 hydrogel at 760, 1146, and 1585 cm^−1^ were revealed by FT-IR, which were related to the C–H, C–N, and C = N stretching vibrations of the imidazole group in ZIF-8 nanoparticles, respectively (Fig. S4) [18]. X-ray photoelectron spectroscopy (XPS) spectra further substantiated the presence of ZIF-8 nanoparticles in the self-strengthening MOFs nanoparticle network hydrogels (Fig. S5) [48]. These results disclose the formation of ZIF-8 nanoparticles and PVA crystalline domains in the PVA/CMC/ZIF-8 hydrogel with MOFs nanoparticle network, which can be served as physical cross-linking sites to strengthen the mechanical properties. Then, as revealed in Fig. 3c, the crystalline domain discrepancies of several hydrogels were visualized by means of atomic force microscopy (AFM) phase diagrams in tap mode. Compared with PVA hydrogel with minimal crystalline features, the introduction of CMC molecules is capable of regulating the crystalline domain distribution of PVA/CMC hydrogel. Furthermore, PVA/CMC/ZIF-8 hydrogel exhibited a prolific distribution of uniformly spaced crystalline domains and abundant MOFs nanoparticles, suggesting that an optimal structural arrangement is conducive to superior mechanical properties. To further quantify the microstructural differences of PVA, PVA/CMC, and PVA/CMC/ZIF-8 hydrogels, wide-angle X-ray scattering (WAXS) and small-angle X-ray scattering (SAXS) measurements were utilized to estimate the average size of crystalline domains and the average distance between the adjacent ones. Two-dimensional WAXS patterns of all hydrogels exhibit homogeneous diffraction rings, indicating that the introduction of CMC induces further crystallization of the polymer network compared to PVA (Fig. 3d). Besides, a significantly enhanced scattering region was observed in WAXS patterns of PVA/CMC/ZIF-8, indicating the formation of a new MOFs nanoparticle network (Fig. 3d, f). As illustrated in Fig. 3f and 2D SAXS patterns with a uniform intensity distribution manifested the isotropic nature of these hydrogels. According to SAXS profiles presented in Fig. 3g and the Bragg equation [49], the average distance (*L*) between adjacent crystalline domains was calculated to be 7.8 nm for PVA and further shortened to 7.3 nm for PVA/CMC. Following the Guinier equation [50], the average size (*D*) of crystalline domains underwent a significant increase from 17.6 nm for PVA to 19.1 nm for PVA/CMC. For the PVA/CMC/ZIF-8 hydrogel, there was only a weak peak, corresponding to a larger structural correlation of ≈20.4 nm, which may be caused by methanol-driven PVA chain aggregation. This decrease in the average inter-domain distance reflects enhanced cross-linking and more robust interactions between domains. Based on the above-mentioned results, it can be concluded that the synergistic interplay of the PVA crystalline domain and the MOFs nanoparticle network that sustains the structural reinforcement and densification of the hydrogel network enables the progressive strengthening mechanical performance of hydrogels.

### Mechanical Properties Modulation of Self-Reinforced MOFs Nanoparticle Network Hydrogel

**Fig. 4:**
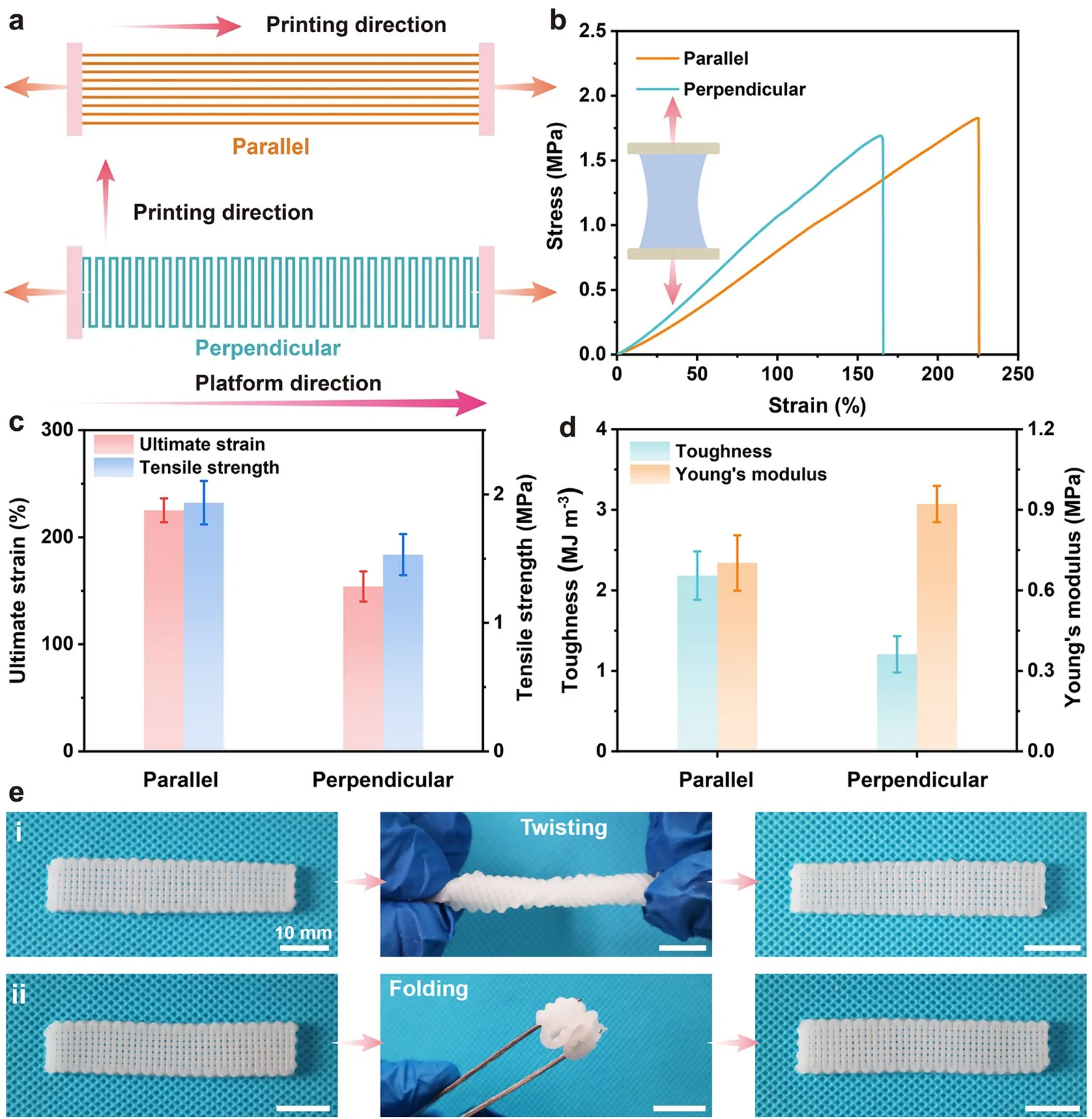
Influence of the printing orientations on mechanical properties of self-reinforcing MOFs nanoparticle network hydrogels. **a** MOFs nanoparticle network hydrogel stripes printed in parallel (top) and perpendicular (bottom) orientations. **b** Tensile stress–strain curves. **c** Tensile strength and ultimate strain. **d** Toughness and Young’s modulus. **e** Photographs of 3D-printed MOFs nanoparticle network hydrogel rectangular grids under various deformations, such as (i) twisting and (ii) folding

It is well known that extrusion printed hydrogels often suffer severe mechanical devastation compared to conventional bulk hydrogels due to defects formed during the printing process or weak adhesion between deposited layers [51]. To explicate this discrepancy in mechanical properties, a rectangular stripe was utilized to explore the influence of printing orientation on the mechanical properties of the 3D-printed MOFs nanoparticle network hydrogels. Specifically, as displayed in Fig. 4a, the printing along the platform deposition direction is called parallel, while the vertical to the platform deposition direction is named perpendicular. It was found that the filament printing orientation displayed a pronounced effect on the mechanical properties of MOFs nanoparticle network hydrogels (Fig. 4b). The fracture strength of MOFs nanoparticle network hydrogels in the perpendicular and parallel directions was 1.53 ± 0.16 and 1.94 ± 0.17 MPa, respectively (Fig. 4c), while the corresponding toughness were 1.21 ± 0.23 and 2.18 ± 0.30 MJ m^−3^, respectively (Fig. 4d), underscoring that the mechanical performance of MOFs nanoparticle network hydrogels printed in the vertical platform deposition direction is weaker than those in the parallel direction. The superior mechanical properties of the latter (parallel direction) may also be related to the tensile bearing capacity of the filament axially oriented enhancement due to parallel printing, which further illustrates the existence of stacking defects and inferior interlayer adhesion between the hydrogel filaments in the perpendicular direction. Additionally, the 3D-printed PVA/CMC/ZIF-8 hydrogel grids with MOFs nanoparticle network can withstand various deformations, such as twisting and folding, and retain structural integrity after stress relief returns to their original shapes or states (Fig. 4e). These results accentuate that the exceptional pliability and mechanical durability of MOFs nanoparticle network hydrogel structures, which in turn have great potential in the construction of functional structures with intricate geometries and biomimetic mechanisms.

**Fig. 5:**
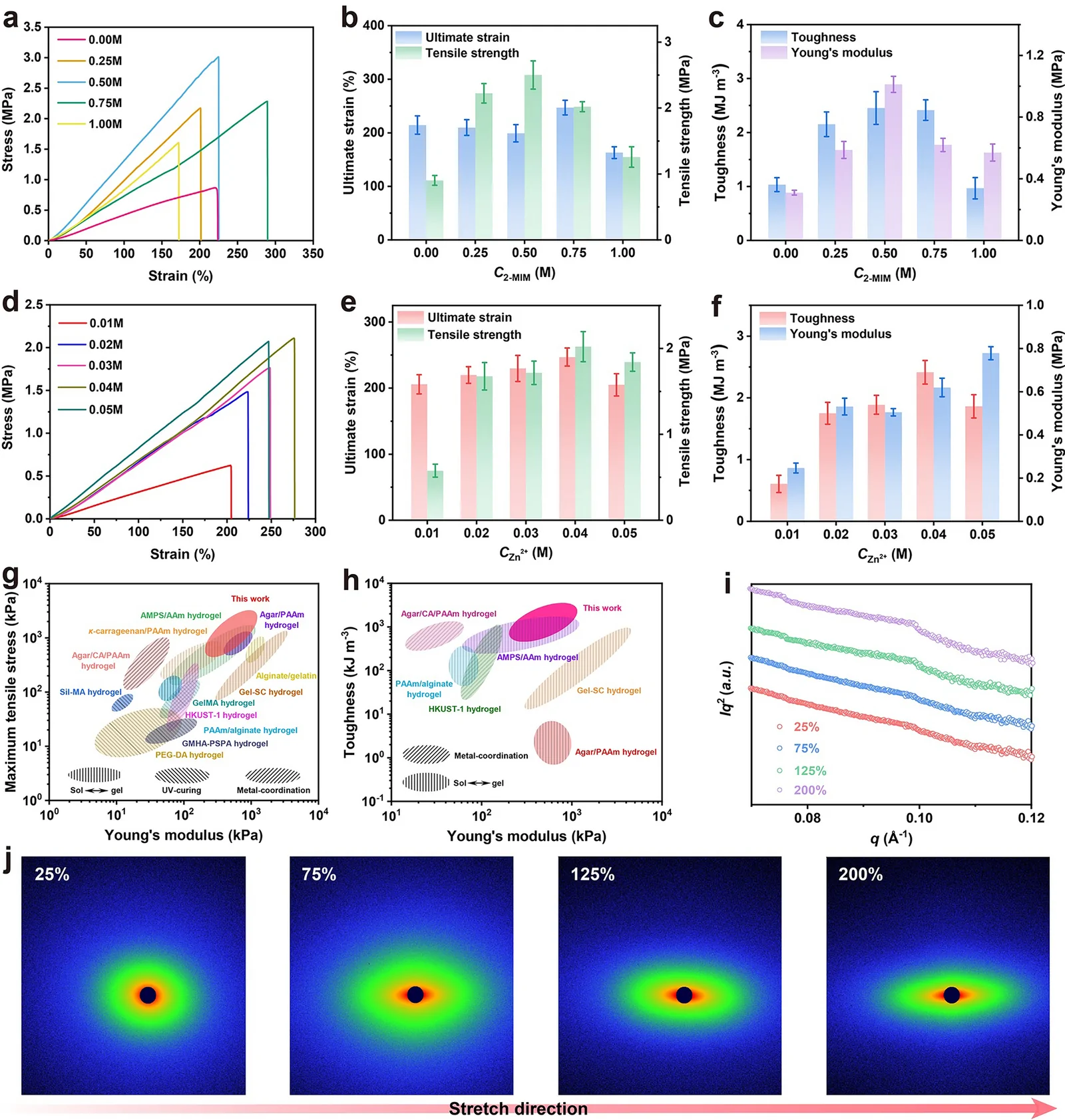
Mechanical properties modulation of self-reinforced MOFs nanoparticle network hydrogel. **a** Representative tensile stress–strain curves of MOFs nanoparticle network hydrogel with different 2-MIM concentrations in inks. **b** Tensile strength and ultimate strain. **c** Toughness and Young’s modulus. **d** Representative tensile stress–strain curves for the effect of Zn^2+^ concentration on mechanical properties. **e** Tensile strength and ultimate strain. **f **Toughness and Young’s modulus. Ashby diagrams of the mechanical properties of MOFs nanoparticle network hydrogels and other ones, including **g** comparison of Young’s modulus and tensile strength as well as **h** comparison of Young’s modulus and toughness. **i** 1D SAXS curves and corresponding **j** 2D SAXS patterns of MOFs nanoparticle network hydrogels under different strains

For MOFs nanoparticle network hydrogels, their mechanical properties are principally affected by the organic ligand components and metal ion concentrations. Accordingly, the influence of organic ligand concentration in precursor inks on the mechanical properties of the hydrogels at a certain concentration of metal solution was first discussed. The fracture strength, toughness, and Young’s modulus of the resulting hydrogel (*i.e.,* PVA/CMC) without 2-MIM in the precursor inks were 0.90 ± 0.07 MPa, 1.03 ± 0.13 MJ m^−3^, and 0.31 ± 0.02 MPa, respectively, which were much lower than those of MOFs nanoparticle network hydrogels (Fig. 5a). This may be due to the presence of only metal coordination network and PVA crystalline domain network in the PVA/CMC hydrogel, resulting in unsatisfactory mechanical properties. When the concentration of 2-MIM raised from 0.25 to 0.75 M, the fracture strength of the MOFs nanoparticle network hydrogels was 2.22 ± 0.14, 2.50 ± 0.21, and 2.02 ± 0.08 MPa, and the toughness was 2.15 ± 0.22, 2.45 ± 0.30, and 2.42 ± 0.19 MJ m^−3^, and the corresponding Young’s modulus was 0.59 ± 0.06, 1.10 ± 0.05, and 0.62 ± 0.04 MPa (Fig. 5b, c). The introduction of 2-MIM leads to higher mechanical properties may be due to the multiple H-bonding interactions of the imidazole ring with -OH on PVA and CMC, while forming ZIF-8 nanoparticles that serve as an additional cross-linking network. However, the breaking strength and toughness of the resulting hydrogels rapidly reduced to 1.26 ± 0.26 MPa and 0.97 ± 0.19 MJ m^−3^ when the 2-MIM concentration was further increased to 1.00 M, which may be due to the diffusion of higher concentration of 2-MIM into the solution during the in situ growth of MOFs nanoparticles, resulting in some MOFs nanoparticles fail to play the role of cross-linking networks in the hydrogel matrix. Subsequently, the effect of metal solution concentration on the mechanical performance of MOFs nanoparticle network hydrogels was examined under a certain concentration of 2-MIM (Fig. 5d–f). When the Zn^2+^ concentration was less than 0.01 M, most of the 2-MIM diffused into the solution during the growth of ZIF-8 nanoparticles, leading to the deterioration of the mechanical properties of MOFs nanoparticle network hydrogels. Nevertheless, with the increase of Zn^2+^ concentration from 0.02 to 0.04 M, the fracture strength of MOFs nanoparticle network hydrogels amplified from 1.68 ± 0.16 to 2.02 ± 0.17 MPa, and the toughness enlarged from 1.75 ± 0.18 to 2.42 ± 0.19 MJ m^−3^. However, when the Zn^2+^ concentration was further increased to 0.05 M, the tensile strength and toughness of the resulting hydrogels declined to 1.84 ± 0.11 MPa and 1.86 ± 0.19 MJ m^−3^, respectively, whereas the elastic modulus raised to 0.78 ± 0.03 MPa. Similarly, the compressive strength of MOFs nanoparticle network hydrogels can be improved from 0.32 ± 0.09 MPa for weak hydrogels to 0.75 ± 0.13 MPa with an optimized MOFs network (Fig. S6). This superior performance is attributed to the incorporation of rigid MOFs nanoparticles that act as additional cross-linking points to create a dense, self-reinforcing network within a hydrogel matrix. Additionally, MOFs nanoparticles often possess surface functional groups that can physically interact with the polymer chains of the hydrogel, resulting in MOFs nanoparticle network hydrogels with exceptional and tunable compression performance. It is worth mentioning that the water content of the MOFs nanoparticle network hydrogels was more than 80%, regardless of the influence of the 2-MIM concentration in the inks or the Zn^2+^ concentration used for the in situ growth of MOFs nanoparticles (Fig. S7). Analogously, these MOFs nanoparticle-reinforced hydrogels exhibited a lower swelling ratio.

Besides, the loading–unloading curves of MOFs nanoparticle network hydrogels have noticeable mechanical hysteresis loops, and the area of the hysteresis loops increases sharply with the increase of strain, indicating superlative energy dissipation characteristics (Fig. S8). After one week of swelling in DI water, the tensile strength of the MOFs nanoparticle network hydrogel declined dramatically from 1.89 ± 0.09 to 0.90 ± 0.01 MPa (Fig. S9a). Likewise, the Young’s modulus and toughness also declined, reaching values of 4.16 ± 0.52 MPa and 0.56 ± 0.01 MJ m^−3^, respectively. Additionally, the swollen PVA/CMC/ZIF-8 hydrogel exhibited lower dissipated energy compared to PVA/CMC/ZIF-8 counterpart (Fig. S9b). This can be attributed to osmotic pressure, which drives the PVA/CMC/ZIF-8 hydrogel to absorb water from the surroundings. The increased hydration of the hydrophilic PVA and CMC chains weakens the hydrogen bonding network, thereby reducing the energy dissipation during mechanical deformation. MOFs nanoparticle network hydrogels also had exemplary fatigue resistance and mechanical stability, which is due to the generation of numerous cooperative H-bonding networks that confer marvelous elasticity (Fig. S10). Subsequently, the mechanical self-recovery performance of the 3D-printed MOFs nanoparticle network hydrogel under various waiting times was evaluated. It was found that the dissipated energy recovery rate was 80.6% after 120 min, indicating that MOFs nanoparticle network hydrogel had satisfactory self-recovery behavior (Fig. S11). Additionally, the recovery process can be divided into three regions, including rapid recovery zone, slow recovery zone, and no recovery zone, which further indicates that the dynamic H-bonding network and metal coordination network in hydrogels progressively recover over time. Concurrently, the optimized 3D-printed MOFs nanoparticles network hydrogel demonstrated a fracture energy of 761.11 J m^−2^ (Fig. S12), suggesting that the synergy of self-strengthening MOFs nanoparticle network and PVA crystalline network can effectively inhibit crack propagation and failure. As shown in Fig. 5g–i, the mechanical properties of 3D-printed self-strengthening MOFs nanoparticle network hydrogels, including tensile strength, Young’s modulus, and toughness, were compared with the sol–gel transition hydrogels, UV-curing 3D printing hydrogels, and metal-coordination 3D-printed hydrogels reported in the literature. For instance, the tensile strain and fracture strength of the 3D-printed MOFs nanoparticle network hydrogels were better than those of lately reported 3D-printed hydrogels (Fig. S13 and Table S1), and their elastic modulus was also superior to other hydrogels (Fig. 5g and Table S2). Simultaneously, the toughness of MOFs nanoparticle network hydrogels was superior to that of the presently reported some hydrogel materials (Fig. 5h and Table S3). Compared with the existing state-of-the-art lubricating hydrogels and meniscus substitute materials, the PVA/CMC/ZIF-8 hydrogel also demonstrated favorable overall properties in terms of fracture strength, toughness, and elastic modulus (Table S4). The results showcase that 3D-printed self-strengthening MOFs nanoparticle network hydrogels possess predominant mechanical properties in terms of fracture strength, elastic modulus, and toughness. We further investigated the reinforcement mechanism behind the MOFs nanoparticle network hydrogels during stretching with SAXS characterization (Fig. 5i, j). Notably, the MOFs nanoparticle network hydrogel transitioned from an isotropic to an oriented structure along the strain stretching direction (Fig. 5j) suggests that stretching induced the alignment of polymer chains in the PVA/CMC/ZIF-8 hydrogel. Such alignment could progressively strengthen the mechanical properties of the hydrogels during the stretching process. Consequently, by taking advantage of the superior mechanical properties of self-strengthening MOFs nanoparticle network hydrogels and the individual manufacturing merits of DIW 3D printing, soft tissue load-bearing structures with high stiffness, strength, and toughness can be constructed.

### Biomimetic Gradient Hydrogel Design and Lubrication Performance Control

**Fig. 6:**
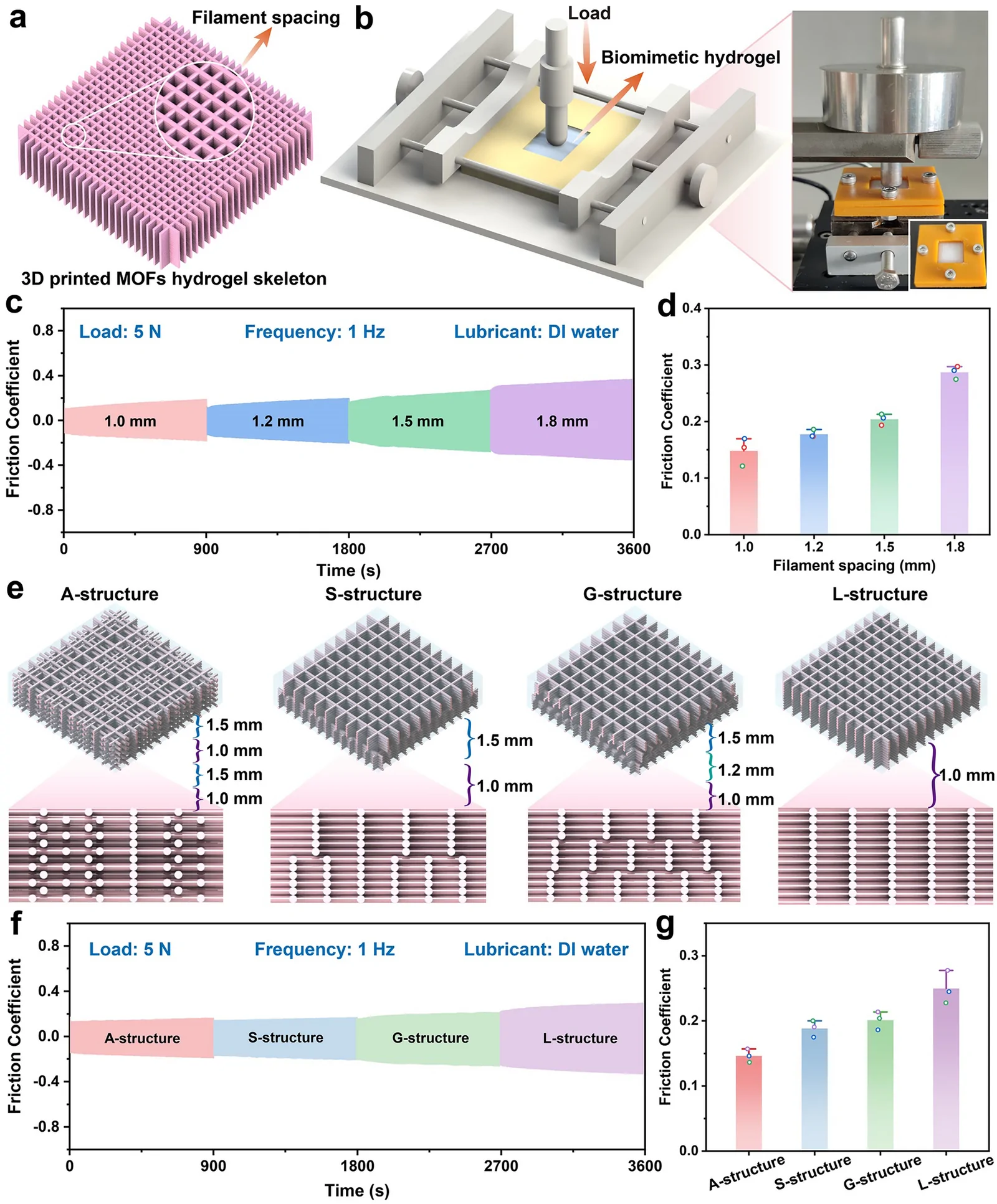
Biomimetic gradient hydrogel design and lubrication performance control. **a** Design of load-bearing skeletons of MOFs nanoparticle network hydrogels with multiple filament spacing. **b** Schematic diagram of the friction test of the biomimetic lubricated hydrogels and the corresponding physical photographs. **c** Friction test curves and **d** corresponding average friction coefficients of biomimetic lubricated hydrogels with different filament spacing. **e** Design of biomimetic gradient lubrication hydrogel with different architectural gradients, spanning A-structure, S-structure, G-structure, and L-structure. **f** Friction test curves and **g** corresponding to the average friction coefficient of biomimetic gradient lubrication hydrogels with dissimilar architectural gradients

For DIW 3D-printed hydrogels, the surface consists of shear-oriented hydrogel filaments that are tightly packed and arranged. Accordingly, it is of great significance to investigate the effect of filament spacing on the lubrication properties of biomimetic gradient hydrogels for comprehending the surface tribological behavior of 3D-printed hydrogels. As shown in Fig. 6a, we designed and fabricated four MOFs nanoparticle network hydrogel structures with filament spacing of 1.0, 1.2, 1.5, and 1.8 mm and integrated them with lubricating PVA/PVP to attain biomimetic lubricating hydrogels with self-reinforcing MOFs nanoparticle network. Afterward, a ball-disk friction testing machine in sliding contact mode was utilized to evaluate the lubrication properties of these biomimetic structured hydrogels (Fig. 6b). As revealed in Fig. 6c, d, tribological tests showcased that the filament spacing of the hydrogel surface enabled by DIW 3D printing has a notable influence on the lubrication performance of biomimetic structured hydrogels. Concretely, when the filament spacing of the 3D-printed MOFs nanoparticle network hydrogel skeletons was 1.0, 1.2, 1.5, and 1.8 mm, the corresponding friction coefficients of the biomimetic lubricated hydrogels were 0.1211, 0.1735, 0.2062, and 0.2903, respectively (Fig. 6d). The biomimetic lubricating hydrogel with small filament spacing displays a relatively low coefficient of friction, which is due to the small contact stress between the friction pair and the hydrogel surface under such load-bearing conditions.

In addition to the impact of filament spacing, we speculate that the gradient load-bearing structure will also have a pronounced effect on the lubrication properties of biomimetic structured hydrogels. To this end, four load-bearing skeletons with different architectural gradients were fabricated by harnessing the same hydrogel ink, including alternating structure (A-structure), sparse-upper and dense-lower structure (S-structure), gradient structure (G-structure), and lattice structure (L-structure), which was adopted to fabricate the biomimetic gradient lubrication hydrogels, as illustrated in Fig. 6e. It is worth mentioning that these structural gradients are substantially constructed by altering the spacing of the printed filaments (in compliance with 1.0, 1.2, and 1.5 mm) between the layers. Subsequently, these hydrogel skeletons with various structural gradients were sequentially ameliorated with self-strengthening MOFs nanoparticle networks and amalgamated with lubricious PVA/PVP hydrogels to fabricate biomimetic gradient lubricated hydrogels. As shown in Fig. 6f and g, the friction coefficients of the biomimetic gradient lubricated hydrogels with A-structure, S-structure, G-structure, and L-structure gradients were 0.1362, 0.1749, 0.2038, and 0.2776, respectively. These results demonstrate that biomimetic gradient hydrogel with alternating structure (A-structure) retains the superlative lubrication performance, because this gradient structure can effectively dissipate stress distribution and restrain structural deformation to reduce the contact stress of the frictional interface under high load-bearing conditions. Furthermore, this dissimilarity in lubrication performance caused by different structural gradients may be due to the dissimilar stress distributions and interfacial contact areas caused by different structural gradients under the identical load. Consequently, this innovative structural gradient design not only showcases superior lubrication properties but also remarkably reinforces the mechanical strength and durability of water-lubricating materials.

### Spatially Engineered Lubricity and Functionality in Architected Biomimetic Gradient Hydrogel Harnessing Multi-Material 3D Printing

**Fig. 7:**
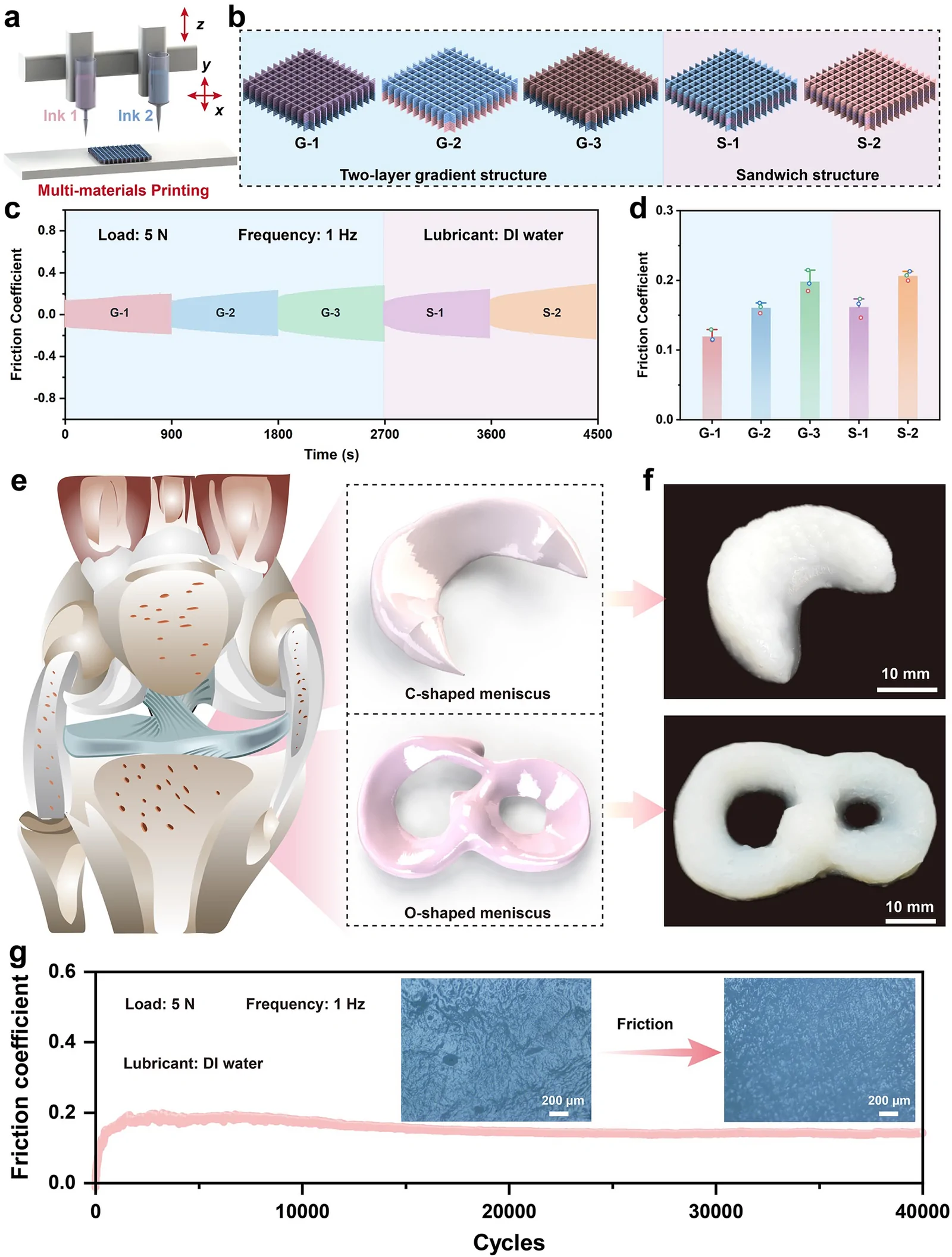
Lubricating performance and applicability of biomimetic gradient hydrogel harnessing multi-material 3D printing. **a** Schematic diagram of biomimetic gradient hydrogel constructed by harnessing multi-material 3D printing. **b** Design of biomimetic gradient lubrication hydrogels with different compositional gradients. **c** Friction test curves and **d** corresponding average friction coefficient of biomimetic gradient lubrication hydrogels with different compositional gradients. **e** Schematic diagram of a human knee joint with meniscus and 3D models of the designed C-shaped and O-shaped meniscus. **f** Photographs of 3D-printed biomimetic gradient slippery hydrogel meniscus with C- and O-shaped configurations. **g** Lubrication persistence and wear resistance of biomimetic gradient slippery hydrogel for 40,000 sliding cycles. Insets are optical microscope images of the surface before and after the hydrogel sliding contact

Over and above harnessing a single material to customize the gradient structure of MOFs nanoparticle network hydrogels, as shown in Fig. 7a, multi-material 3D printing can also be adopted to engineer biomimetic gradient lubricated hydrogels with dissimilar compositional gradients. For this purpose, five MOFs nanoparticle network hydrogel load-bearing skeletons with various compositional gradient configurations were contrived by integrating multi-material 3D printing and two hydrogels with different mechanical properties (Fig. 7b), including three double-layer gradient structures (G-1, G-2, and G-3) and two three-layer sandwich structures (S-1 and S-2). For the G-1, G-2, and G-3 gradient hydrogel load-bearing skeletons, the layer thickness of these structures is 12 layers in total, that is, a six-layer structure was printed with one component (ink 1 or ink 2) at the bottom, and then, six-layer structure was printed with another component (ink 1 or ink 2) on top of it. For the S-1 and S-2 gradient hydrogel skeletons, furthermore, the bottom and top four layers were constructed with the alike hydrogel ink, while the middle four layers were created with another hydrogel ink. Subsequently, these MOFs nanoparticle network self-reinforced 3D-printed hydrogel architectures were combined with lubricative PVA/PVP through mechanical interlocking to enable biomimetic gradient lubricating hydrogels with diverse compositional gradients. As depicted in Fig. 7c and d, under the conditions of 5 N load and 1 Hz frequency, the lubrication properties of these biomimetic gradient lubricated hydrogels were investigated by leveraging glass spheres as the friction pair and DI water as the lubricant, and the friction coefficients of the biomimetic hydrogels with G-1, G-2, G-3, S-1, and S-2 structures were 0.1141, 0.1528, 0.2147, 0.1733, and 0.2129, respectively. The biomimetic gradient hydrogel with G-1 structure showcased better lubricating properties, which may be due to the load-bearing capacity of G-1 structure is better than that of G-2 and G-3 ones. Compared with the S-2 structure, the biomimetic gradient lubricating hydrogel with the S-1 configuration also possessed superlative lubricating properties. The difference in lubrication performance under the identical load-bearing condition is mainly owing to the different stress distribution and contact areas caused by various gradient structures, which is conducive to the hydrogel to resist elastic deformation and improve lubricity.

As is known to all, meniscus is a pair of fibrotic cartilage pads in the knee joint, which is of particular importance in various biomechanical functions [52], including protecting cartilage, lubricating cartilage, and stabilizing the knee joint (Fig. 7e). For meniscus injuries, however, it is difficult to achieve artificial meniscus substitutes with biomimetic structures and lubrication functions leveraging conventional materials and techniques. Surprisingly, multi-material 3D printing techniques and slippery-structured hydrogels offer an auspicious route for constructing meniscus alternatives with similar geometries, exceptional mechanical properties, and bio-lubrication capabilities to natural meniscus. For this purpose, 3D digital models of two types of knee meniscus, such as the C-shaped and O-shaped meniscus, were created by modeling software (Fig. 7e). Subsequently, two biomimetic gradient hydrogel structures with meniscus shapes were created by multi-material 3D printing, and two slippery hydrogel meniscus alternatives with biomimetic gradient were obtained after MOFs nanoparticle network reinforcement and lubricated PVA/PVP hydrogel coupling (Fig. 7f). The elaborately constructed biomimetic slippery hydrogel meniscus exhibits clear meniscus shape characteristics. Furthermore, the lubrication durability and wear resistance of the biomimetic gradient hydrogel meniscus were further evaluated, and the exemplary lubricity was found to be maintained after 40,000 long-term frictions (Fig. 5g). Optical microscopy images of the hydrogel surface before and after sliding contact further revealed their meritorious abrasion resistance. The above results substantiate that the association of multi-material 3D printing technique and lubricious hydrogel strengthened with MOFs nanoparticle network holds the potential to create tailor-made biomimetic hydrogel meniscus alternative with high strength, superior lubricity, and abrasion resistance.

## Conclusion

Inspired by the stiff gradient structure of the soft–hard tissue interface, a biomimetic gradient hydrogel with soft lubricated surface and hard load-bearing skeleton was fabricated combined with the 3D-printed biomimetic gradient MOFs nanoparticle network architecture and lubricious PVA/PVP hydrogel, in which the 3D-printed gradient skeleton could be served as the load-bearing phase to counter deformation, while the soft hydrogel lubricating phase was leveraged to maintain superior lubrication performance. Additionally, the mechanical performance of self-strengthening MOFs nanoparticle network hydrogels can be tuned by altering the composition of organic ligands in hydrogel inks and the concentration of metal ions utilized for MOFs nanoparticles growth. Numerous biomimetic lubricating hydrogels with structural and compositional gradients were constructed to regulate their lubrication properties by manipulating the composition of hydrogel inks and the structural design of 3D printing, underscoring the efficacy of the biomimetic design in providing superior lubrication. As a proof-of-concept, two slippery hydrogel meniscus alternatives with complicated gradient structures, reliable cushioning layers, and anti-friction lubrication were constructed by leveraging multi-material 3D-printed MOFs nanoparticle network hydrogels and lubricious PVA/PVP hydrogels. This work offered an innovative avenue for the development of novel biomimetic structured water-lubricated materials with exemplary load-bearing capacities and lubrication properties and exhibited significant application prospects in the field of soft tissue-bearing alternative materials.

## Experimental Section

Specific experimental protocols and characterization methods are provided in the Supporting Information.

## Supplementary Information

Below is the link to the electronic supplementary material.

Supplementary file1 (DOCX 2988 KB)
