# Supplementary material for: Biomimetic Gradient Lubrication Hydrogel Contrived by Self-Reinforced MOFs Nanoparticle Network
Source: Nanomicro Lett. 2026 Jan 12;18:150. doi: 10.1007/s40820-025-02001-x (PMC12791084; doi:10.1007/s40820-025-02001-x)
Supplement: Supplementary file 1 — Supplementary file1 (DOCX 2988 KB) [file 40820_2025_2001_MOESM1_ESM.docx]

Supporting Information for

**Biomimetic Gradient Lubrication Hydrogel Contrived by Self-Reinforced MOFs Nanoparticle Network**

Desheng Liu^1^, Yixian Wang^2^, Changcheng Bai^1^, Danli Hu^1^, Xingxing Yang^2^, Yaozhong Lu^1^, Tao Wu^1,^ ^3^, Fei Zhai^3^, Pan Jiang^1^*, Xiaolong Wang^1^*, Weimin Liu^1^

^1^ State Key Laboratory of Solid Lubrication, Lanzhou Institute of Chemical Physics, Chinese Academy of Sciences, Lanzhou 730000, P. R. China

^2^ School of Chemistry and Chemical Engineering, State Key Laboratory Incubation Base for Green Processing of Chemical Engineering, Shihezi University, Shihezi 832003, P. R. China

^3^ Shandong Laboratory of Advanced Materials and Green Manufacturing at Yantai, Yantai Zhongke Research Institute of Advanced Materials and Green Chemical Engineering, Yantai 264006, P. R. China

*** Corresponding authors. E-mail: [pan.jiang@ijm.fr](mailto:pan.jiang@ijm.fr) (Pan Jiang); [wangxl@licp.cas.cn](mailto:wangxl@licp.cas.cn) (Xiaolong Wang)

**S1 Experimental Section**

**S1.1 Materials and Reagents**

Polyvinyl alcohol (PVA, 99% hydrolyzed, DP = 1750±50) and Polyvinylpyrrolidone K-15 (PVP K-15, M.W. 8000, 98%) was purchased from Sinopharm Chemical Reagent Co., Ltd. Carboxymethylcellulose sodium (CMC, USP Grade, viscosity of 5000~15000mPa·s) was purchased from Macklin. 2-Methylimidazole (2-MIM, 98%) and Zinc nitrate hexahydrate (Zn (NO_3_)_2_·6H_2_O, 99%) were purchased from Shanghai Aladdin Biochemical Technology Co., Ltd. Ultrapure water (*ρ* = 18.25mΩ·cm) was purified in the laboratory. All chemical agents were directly used without any purification.

**S1.2 Preparation of the Precursor Inks**

The PVA powder was dissolved in distilled water at 95 °C for 2 h to obtain a transparent PVA aqueous solution (12.5 wt%). Subsequently, dispersions with different 2-MIM content were prepared by incorporating 2-MIM at molar concentrations (relative to the water content in PVA aqueous solution) of 0.25, 0.50, 0.75 and 1.00 M in 40 g of PVA aqueous solution. The CMC powders, in mass ratios of 4.0 wt% with respect to the PVA aqueous solution, were then added into the PVA/2-MIM dispersions and blended using a planetary speed-mixer (DAC 150 FVZ-K, Synergy Devices Ltd) at 3000 rpm for 2 min to achieve the rheological properties required for direct ink writing (DIW). The prepared four kinds of solid-like precursor inks were labeled as PVA/CMC-*X*, where *X* refers to the molar concentration of 2-MIM in the precursor mixture. The hydrogel precursor inks were stored in the refrigerator for 24 h for complete hydration. As a control, PVA/CMC ink was prepared through similar procedures except that the 2-MIM was not added to the PVA aqueous solution.

**S1.3 Three-Dimensional Printing of the Precursor Inks and** **Postprocessing**

The different precursor inks were first transferred into a 10 mL cartridge equipped with a stainless-steel nozzle (21 G, 510 μm) and centrifuged at 8000 rpm for 5 min to remove air bubbles. The syringe was then mounted on a commercial 3D bioprinter (3D Bio-Architect Sparrow, Regenovo, China) and pneumatically extruded onto the pre-cooling platform (-5 °C) in a layer-by-layer fashion. Printing path is controlled by customized G-code. Specifically, the printing parameters of thin film samples (10 mm × 10 mm) for mechanical testing are as follows: the extrusion pressure of 0.3 to 0.5 MPa, the moving speed of 5 to10 mm s^-1^, layer thickness of 400 μm, and the distance between two adjacent deposited filaments was 600 μm. Furthermore, a PVA/CMC-0.75 precursor ink was used to fabricate hydrogel constructs with various architectures under the same print settings operation, except that the interval between the deposited filaments was set to 1.2 mm. All STL files of 3D structures were generated by *Solidworks* software and converted to G-code prior to 3D printing. The 3D printing procedures for all structures were carried out at room temperature. Resulted printed constructs were then subjected to thrice freezing-thawing cycles to obtain white and opaque hydrogels (PVA/CMC) that feature good mechanical properties, in which freezing in the refrigerator at -20 °C for 6 h and subsequent thawing at ambient temperature at 25 °C for 6 h.

**S1.4 Controlled In Situ Growth of ZIF-8 Nanoparticle with Post-Printing Treatment**

The printed PVA/CMC hydrogel objects were immersed in the methanolic solutions of zinc nitrate hexahydrate with different concentrations (10 mM, 20 mM, 30 mM, 40 mM, and 50 mM) for 24 h at room temperature to initiate in situ growth of ZIF-8 nanoparticles while chelating carboxylic groups in the CMC, which would further reinforce the mechanical strength of the printed constructs. After that, the printed constructs were immersed in distilled water for solvent exchange equilibrium prior to proceeding with the mechanical testing. The finally obtained 3D printed MOFs nanoparticle network hydrogels were named PVA/CMC/ZIF-8.

**S1.5 Fabrication of Biomimetic Gradient Lubrication Hydrogel**

To manufacture constructs with architectural gradients, three separate square models (alternating structure (A-structure), sparse-upper and dense-lower structure (S-structure), and gradient structure (G-structure) with dimensions of 15 mm (length) × 15 mm (width) × 5 mm (height) was programmed by *Solidworks* software and transformed into G-code for print paths. A PVA/CMC-0.75 ink was loaded into a transparent syringe equipped with a stainless-steel nozzle (21 G, 510 μm) and centrifuged at 10000 rpm for 5 min to eliminate air bubbles. All the structures are deposited at a filament extrusion speed of 5 mm s^-1^ and under an applied pressure of 0.45 MPa. The filament spacing of the entire gradient constructs varied gradually from 1.0 to 1.5 mm. Each layer was deposited to the underlying layer perpendicularly to form a 0° or 90° strut structure. Ultimately, various gradient hydrogel scaffolds with a height of 12 layers were fabricated. As a control, a lattice structure (L-structure) with a fiber spacing of 1.0 mm also was manufactured in a similar manner.

Furthermore, a multi-materials DIW was used to manufacture compositional gradient hydrogel constructs with various structures. Briefly, take the PVA/CMC-0.25 and PVA/CMC-1.00 inks as an example. The PVA/CMC-0.25 and PVA/CMC-1.00 inks were filled into two cartridges, respectively. After the air bubbles are removed, the two inks are successively squeezed out onto a low-temperature platform in a layer-by-layer manner. Six layers of PVA/CMC-0.25 ink was printed firstly, then the six layers of PVA/CMC-1.00 ink was printed on the top to obtain a G-1 scaffold with a “two-layer” gradient structure. Conversely, a twelve-layer G-2 gradient hydrogel scaffold can be fabricated in a similar manner. Besides, for G-3 scaffold, the bottom and top six layers can be made from PVA/CMC-1.00 and PVA/CMC inks, respectively. The PVA/CMC, PVA/CMC-0.25, and PVA/CMC-1.00 inks were used to construct S-1 and S-2 gradient hydrogel constructs with sandwich structures. The PVA/CMC ink was used to print the middle four layers of the S-1 and S-2 gradient hydrogel constructs. The PVA/CMC-1.00 ink was mainly printed as the bottom four layers and the top four layers of the S-1 gradient hydrogel construct. And meanwhile, for the S-2 gradient hydrogel construct, the bottom four layers and the top four layers were made of the PVA/CMC-0.25 ink. During the printing process, the needle diameter, layer thickness, printing speed and extrusion pressure used for the construction of the compositional gradient hydrogel supports were maintained constant at 0.51 mm, 0.40 mm, 5 mm s^-1^ and 0.45 MPa, respectively. The distance between two adjacent deposited filaments was set to 1.2 mm.

The freezing-thawing experiment procedures for post-treatment of gradient hydrogel constructs were the same as mentioned above. The printed biomimetic gradient PVA/CMC hydrogel objects were immersed in the methanolic solutions of zinc nitrate hexahydrate (50 mM) for 24 h at RT for in situ growth of ZIF-8 nanoparticles while ion crosslinks the carboxylic groups in the CMC. After that, various 3D printed PVA/CMC/ZIF-8 biomimetic gradient structures were placed in a 35 mm Petri dish with a 15 wt.% polymer solution consisting of 10 wt.% PVA and 5 wt.% PVP, flattened with a counterpoise (5 g). Subsequently, the hydrogel samples were frozen at -20 °C for 6 h and thawed at 25 °C for 6 h, which was repeated five cycles to obtain bionic gradient lubricated hydrogels.

**S1.6 Fabrication of Slippery Hydrogel Meniscus-like Constructs**

To print the meniscus, a structure was designed by *Solidworks* software and converted into G-code for the print paths. By using the same printing parameters, a meniscus-like construct was printed using PVA/CMC-1.00 (bottom layers) and PVA/CMC (top layers) hydrogel precursor inks. The post-processing of the 3D printed meniscus-like constructs was carried out using the same procedure as mentioned above. The 3D printed PVA/CMC/ZIF-8 meniscus-like construct was immersed to a 15 wt.% polymer solution, composed of 10 wt.% PVA and 5 wt.% PVP. Subsequently, the samples were subjected to five cycles of freezing at -20 °C for 6 h and thawing at RT for 6 h to achieve slippery hydrogel meniscus-like constructs.

**S1.7 Rheology Behavior**

The printability of all hydrogel inks was assessed through a rotational rheometer (HAAKE, RS6000, Germany) with a diameter of 35 mm parallel plate geometry and a gap distance of 1 mm. All hydrogel inks were centrifuged to remove air bubbles prior to rheological measurement. All measurements were carried out at 25 °C with a frequency of 1 Hz. Shear-thinning behavior of the as-synthesized inks were evaluated by steady-state flow sweep in the shear rate range from 0.01 to 100 s^-1^. The loss modulus (G”) and storage modulus (G’) of the inks were preliminary measured by oscillation stress sweep at a frequency of 1 Hz to assess its self-supporting ability, and the shear stress amplitude ranged from 1 to 10000 Pa. The yield stress (*τ_y_*) of the inks was defined as the stress value applied at the intersection of G′ and G" (G′ = G"), which indicates the occurrence of plastic deformation. Alternate step shear rate sweeps were carried out to determine the thixotropic viscosities of the inks between a low shear rate (0.01 s^-1^) and a high shear rate (60 s^-1^) for 200 s each one. Alternate oscillatory shear stress sweeps were performed to explore the thixotropic moduli (G′/G″) recovery behavior of the inks. The alternating shear stresses were switched from a low stress (10 Pa) to subsequent a high stress (15000 Pa) with 200 s retention time interval between each shear stress stage.

**S1.8 Evaluation of Lubrication Performance**

The frictional behavior of the 3D printed MOFs particle network hydrogels with biomimetic gradient structure was evaluated by employing a conventional reciprocating tribometer (CSM Co. Ltd., Switzerland) with ball-on-disk configuration. The hydrogel samples (15 mm × 15 mm × 5 mm) were clamped into a self-designed mold, and sufficient deionized water was added to the surface of the hydrogels as a lubricant. The contact pair was a glass sphere with a radius of 3 mm. In the sliding friction tests, the sliding speed was 1.57 cm/s, and the sliding path was 5 mm at constant frequency of 1 Hz. Furthermore, the influence of the applied load and sliding frequency on the friction coefficient was verified by the univariate factor method, in which the normal force was 1.0-10.0 N and the rotation frequency was 1-5 Hz. The coefficient of friction (COF) was recorded under various experimental conditions. For each sample, the test duration was conducted for 900 s. The tribological tests were repeated at least three times under each condition to ensure the reliability and repeatability of the obtained COF.

**S1.9 Characterization**

***Tensile Properties***

The mechanical properties of the 3D printed MOFs nanoparticle network hydrogels were investigated using an electronic universal testing machine (EZ-Test, SHIMADZU) equipped with a maximum force of 500 N loading cell. The hydrogel tensile specimens were printed into rectangular shapes with dimensions of 40 mm (*L*) × 8 mm (*W*) × 1 mm (*t*) and stretched monotonically at the speed of 100 mm min^-1^ until failure of the sample. The tensile stress-strain curves were recorded. During the whole experiment, all the hydrogel specimens were coated with a layer of perfluoropolyethers oil (PFPE, Sigma Aldrich) to prevent the dehydration of samples. The tensile strain (*ε_b_*, %) was defined as the change in the deformation relative to the initial length of the undeformed specimen. The normal tensile strength (*σ_b_,* MPa) was obtained by the maximum tensile force divided by the cross-sectional area of the undeformed specimen. The elastic modulus (*E*, MPa) was defined as the slope of the stress-strain curve in the linear elastic region between 10 and 20% strain. Moreover, the tensile toughness (*T*, MJ m^-3^) of the hydrogel was estimated from the integrated area under the stress-strain curve.

For the fatigue resistance of the 3D printed MOFs nanoparticle network hydrogel, ten consecutive loading-unloading cycles were repeatedly performed at a crosshead speed of 100 mm min^-1^ under the same strain (100%), while no time intervals were introduced between successive cycles. To investigate its hysteresis effect under deformation, the loading-unloading cycles measurements under various strains (25%-150%) were also conducted. The dissipated energy (*U_hys_*) for each cycle can be estimated by integrating the area of the stress-strain curves hysteresis loop.

To investigate the self-recovery capability of the 3D printed MOFs nanoparticle network hydrogels after deformation, the hydrogel samples were first extended to a strain of 100% and then unloaded at the speed of 100 mm min^-1^. Then, the hydrogel was allowed to stand for different time intervals in an unstretched state between two consecutive stretching cycles for recovery. The recovery rate (%) is defined as the ratio of the energy dissipated (*U_hys_*) at different recovery times to that of the first loading cycle.

***Wettability Characterization***

Water contact angles of 3D printed MOFs nanoparticle network hydrogel at ambient temperature were measured on a drop shape analyzer (KRÜSS DSA100, Germany). In a typical case, a 5 μL of water droplet was carefully deposited onto the airside surface of the hydrogel sample, and the static contact angle was seized after 5 s. The mean contact angle was obtained from the results of at least three measurements.

***XPS Measurements***

The surface elemental composition of the hydrogel samples was characterized by X-ray photoelectron spectroscopy (XPS, ESCALAB 250Xi, Thermo Fisher, USA) with an Al K*α* radiation source. All the spectra were calibrated to the binding energy of the adventitious C 1s peak at 284.8 eV.

***ATR -FTIR Measurements***

Attenuated total reflection Fourier transform infrared spectroscopy (ATR-FTIR) (Nicolet iS50, Thermo Fisher Scientific, USA) were performed to characterize the characteristic functional groups of the hydrogel specimens. The hydrogel spectra were recorded in wavenumber range of 4000-600 cm^-1^ with a resolution of 4 cm^-1^ scans. Before the tests, the hydrogel samples were freeze-dried in a vacuum.

***Differential Scanning Calorimetry (DSC) Measurements***

In the differential scanning calorimeter (DSC, STA449F3, NETZSCH, Germany) test, the air-dried samples were heated up from 50 ℃ to 250 ℃ at a heating rate of 10 ℃ min^-1^ under a nitrogen flow of 30 mL min^−1^.

***X-ray Diffraction (XRD) Measurements***

The formation of crystal phase in 3D printed MOFs nanoparticle network hydrogel was determined by X-ray diffraction (XRD, Rigaku Smartlab, Japan) with Cu K*α* radiation (*γ* = 1.5406 Å).

***SEM Observation***

To depict the microstructures within the fabricated 3D printed MOFs nanoparticle network hydrogels, morphology observation was conducted on a Scanning Electronic Microscopy (SEM, Phenom ProX, Netherlands) at an acceleration voltage of 10-15 kV. All hydrogel samples were freeze-dried, and then the surfaces of the lyophilized sample were sputter-coated with a layer of gold using an ion-sputtering apparatus (GVC-1000, China).

***SAXS and WAXS Measurements***

Small-angle X-ray scattering (SAXS) and wide-angle X-ray scattering (WAXS) measurements were performed by leveraging the Xeuss 3.0 SAXS/WAXS to characterize the microstructure of hydrogels. The detector model is Eiger2R 1M, with a pixel edge length of 75 μm, and uses a copper target 8.05 KeV X-ray with a wavelength of 1.54189 Å. The test environment is vacuum (< 1 mbar) and the distance from the detector to the sample is 650 mm. The average distance (L) between adjacent crystalline domains was calculated by Bragg’s equation:

$L=\frac{2\pi}{q_{max}}$ (S1)

where *q*_max_ corresponds to the peak position of 1D SAXS profile. According to the reciprocal principle, the scattering curves in the low *q* region correspond to the large scale.

***AFM Observation***

The microphase separation structure of the hydrogel sample was measured by an atomic force microscope (AFM, JPK Nanowizard 4XP Germany) in MPP rotating cantilever tapping mode with a silicon probe with a tip radius of curvature of 7.8 nm, a force constant of 0.4546 N/m, and a scanning speed of 2 Hz.

**Supplementary Figures and Tables**


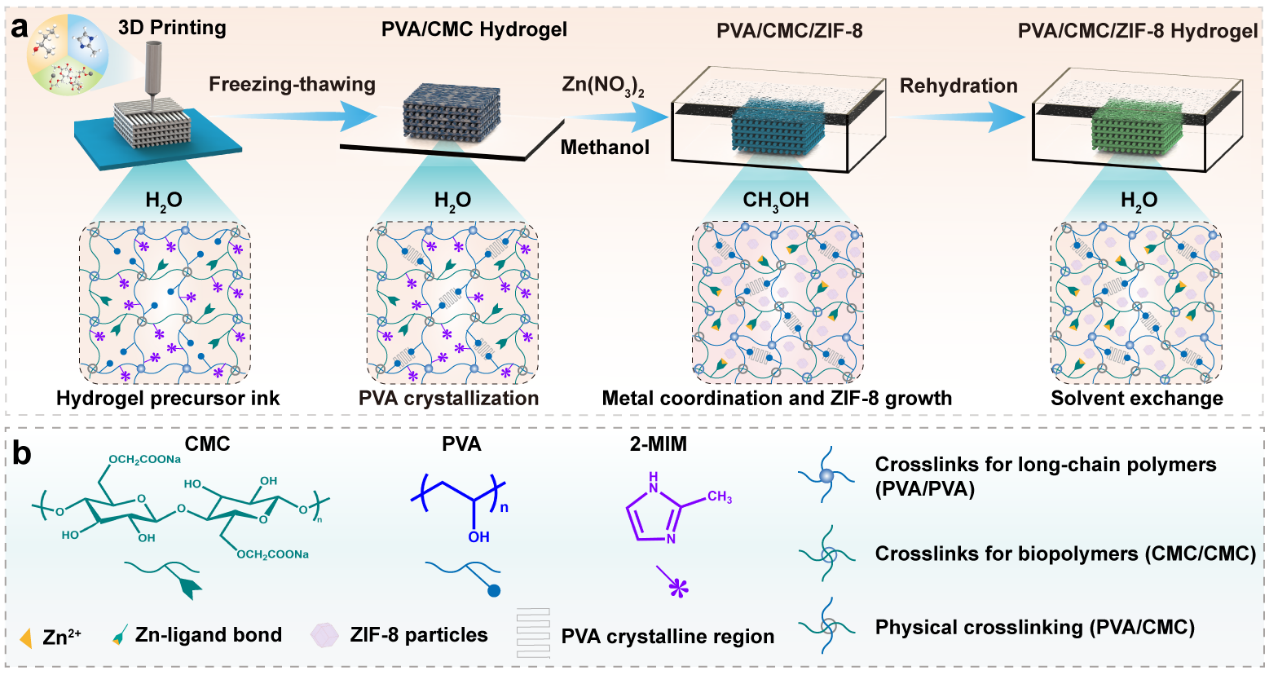


**Fig. S1** Schematic illustrating the fabrication of 3D printed MOFs nanoparticle networks hydrogel. The composite inks composed of PVA, CMC, and 2-MIM are 3D printed into designed patterns first, and then undergoes freezing-thawing, in situ growth of MOFs nanoparticles, and rehydration process transform them into 3D printed MOFs nanoparticle networks hydrogel.


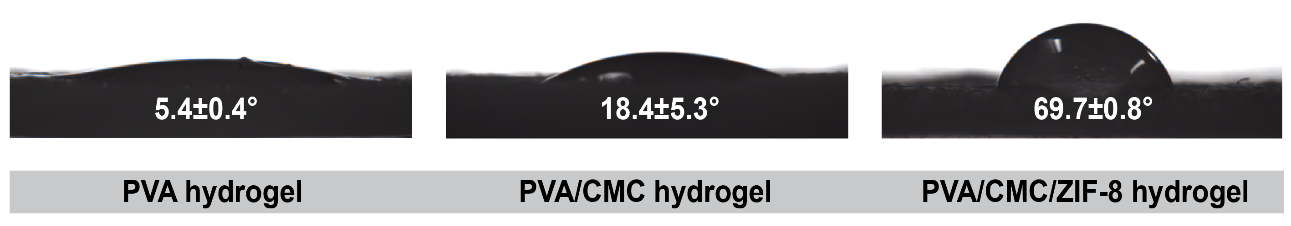


**Fig. S2** Water contact angles of PVA, PVA/CMC, and PVA/CMC/ZIF-8 hydrogels


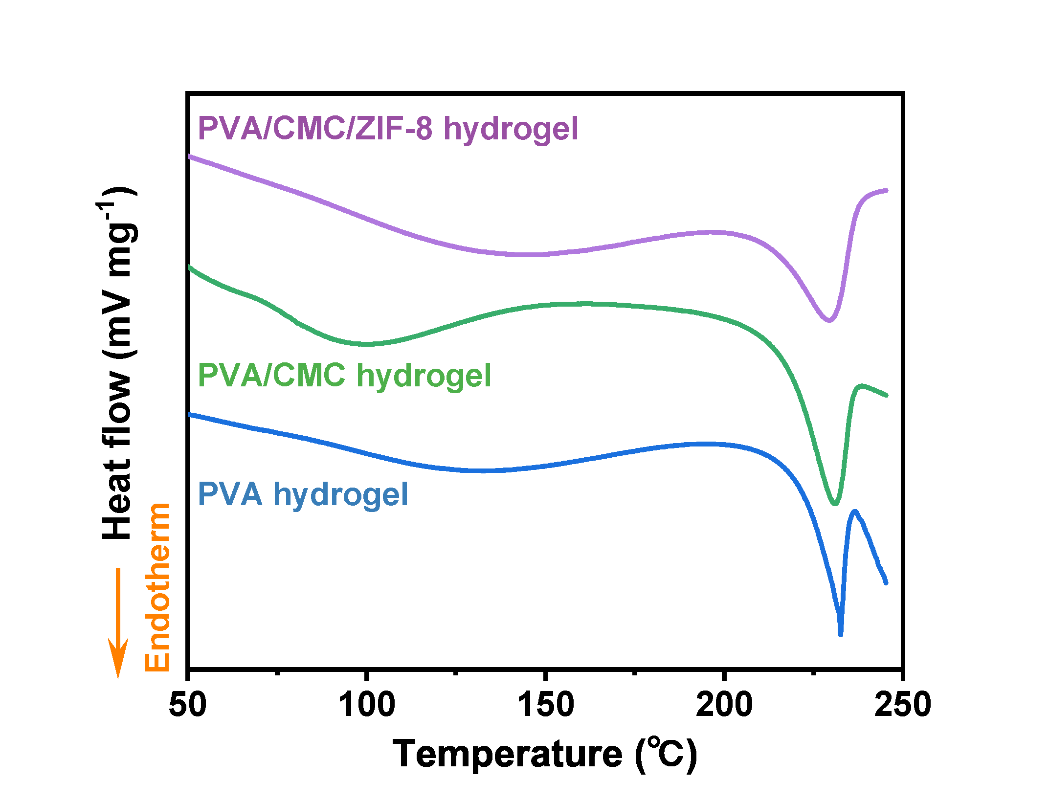


**Fig. S3** DSC curves of PVA, PVA/CMC, and PVA/CMC/ZIF-8 hydrogels


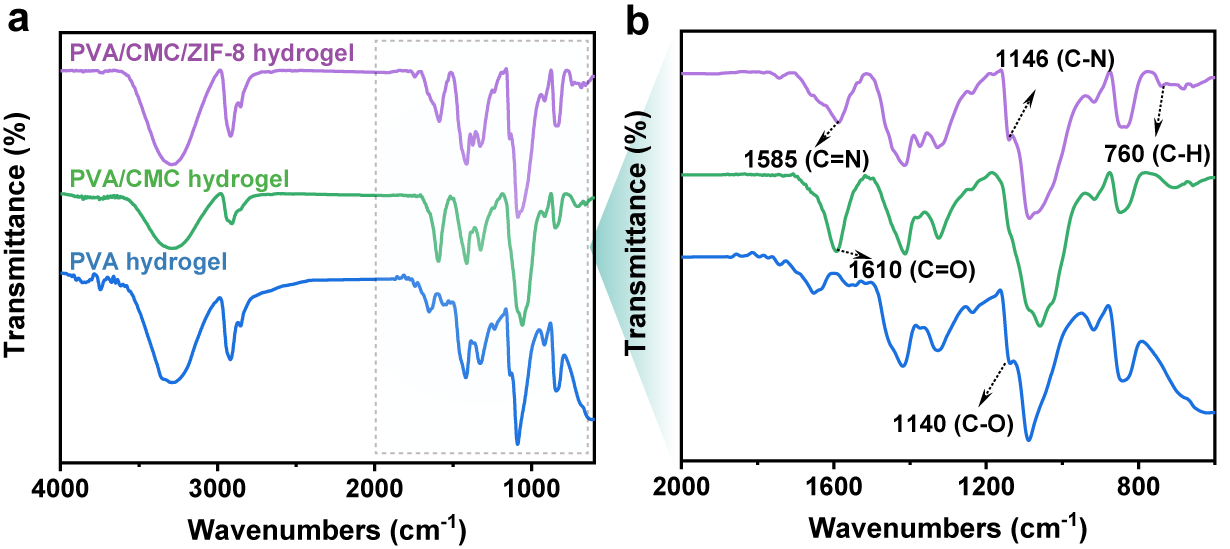


**Fig. S4** FT-IR spectra of PVA, PVA/CMC, and PVA/CMC/ZIF-8 hydrogels


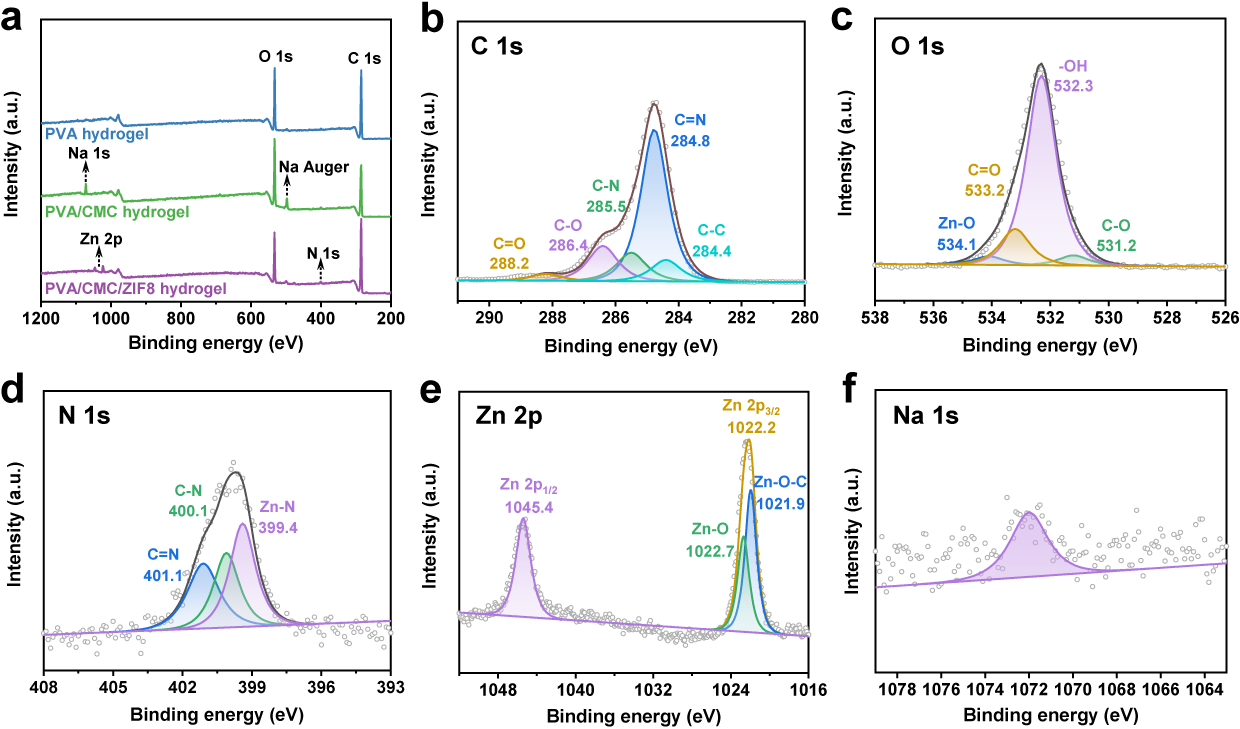


**Fig. S5** (**a**) XPS full spectra of PVA, PVA/CMC, and PVA/CMC/ZIF-8 hydrogels. High-resolution (**b**) C 1s, (**c**) O 1s, (**d**) N 1s, (**e**) Zn 2p, and (**f**) Na 1s spectra of PVA/CMC/ZIF-8 hydrogel


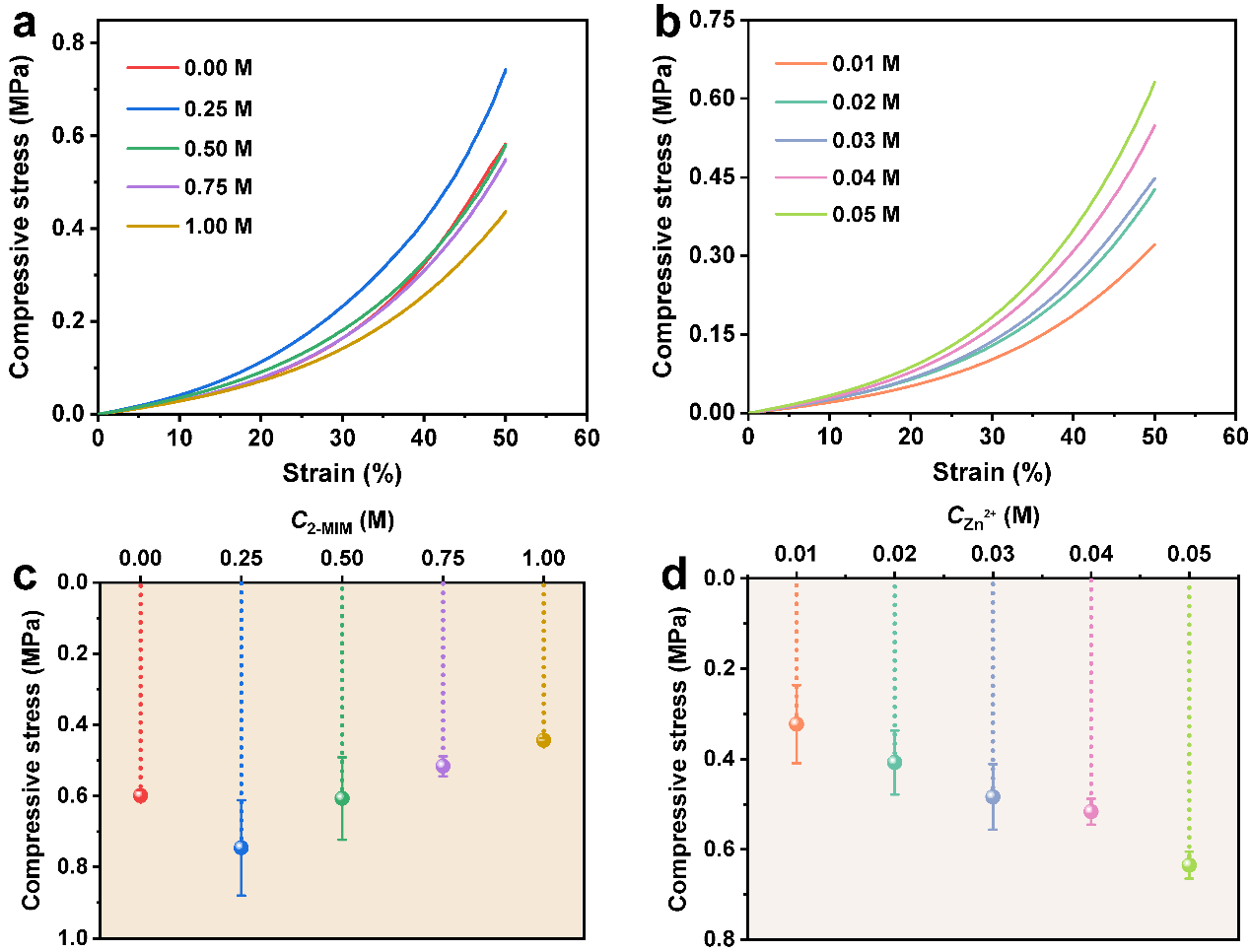


**Fig. S6** Compression performance of hydrogel with MOFs nanoparticle self-reinforcing network. (**a**) Representative compressive stress-strain curves and the corresponding (**c**) compressive strength of MOFs nanoparticle network hydrogel with different 2-MIM concentration in ink. (**b**) Representative compressive stress-strain curves and the corresponding (**d**) compressive strength for the effect of Zn2+ concentration on mechanical properties


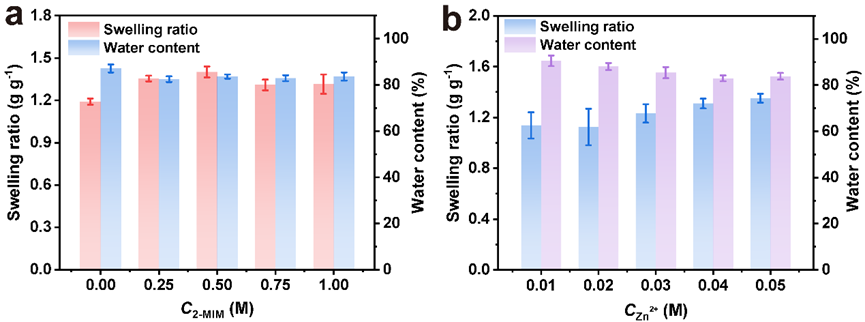


**Fig. S7** Water content and swelling ratio of MOFs nanoparticle network hydrogel. (**a**) The effect of 2-MIM concentration in ink on water content and swelling ratio. (**b**) The effect of Zn^2+^ concentration on water content and swelling ratio


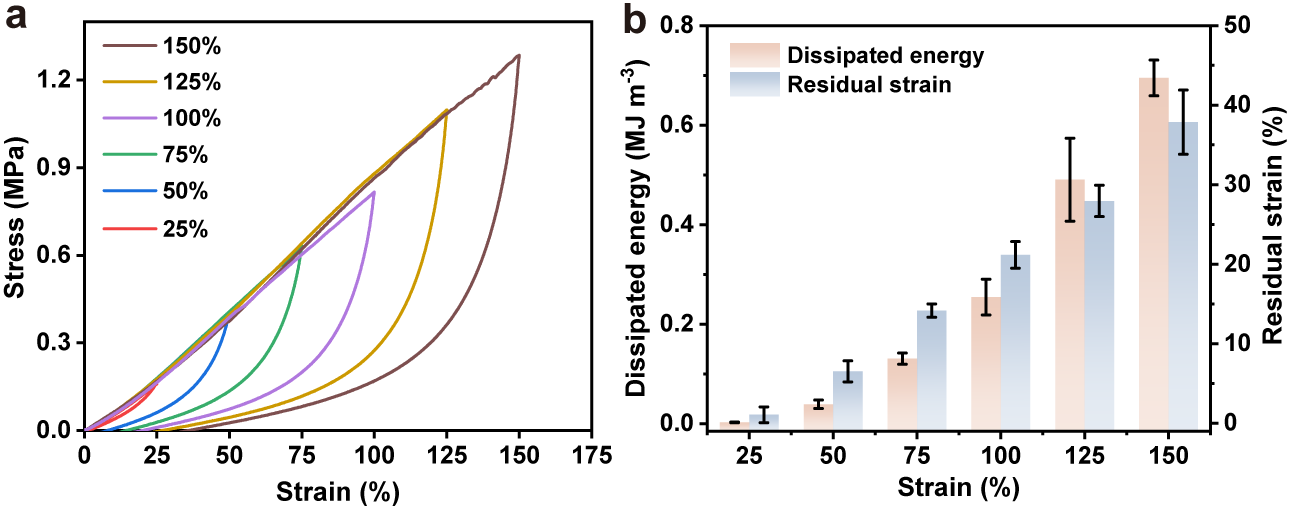


**Fig. S8** Energy dissipation mechanism of MOFs nanoparticle network hydrogels. (**a**) Loading-unloading curves of hydrogels under different strains. (**b**) Dissipated energy and residual strain of hydrogels under different strains


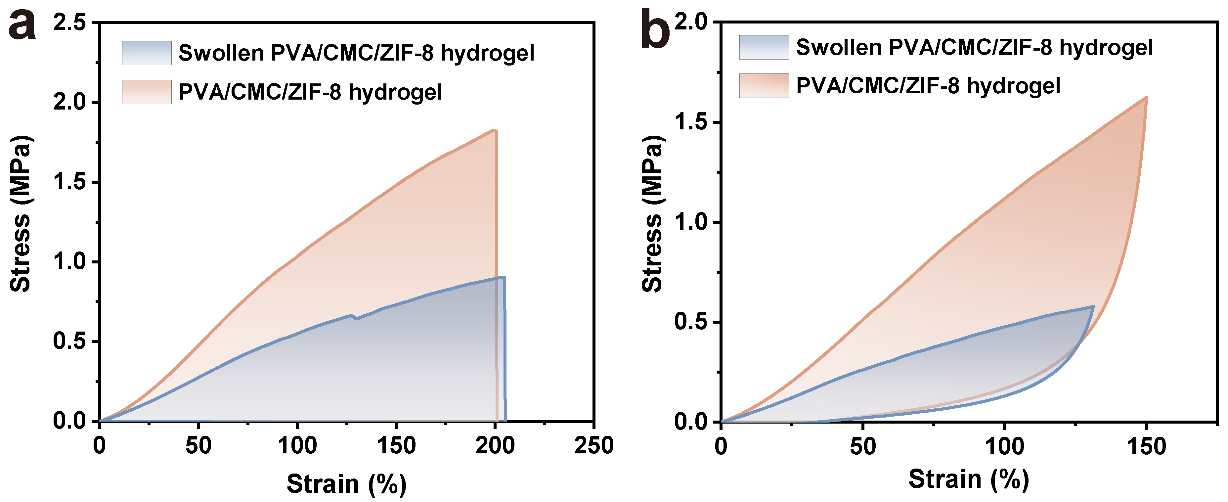


**Fig. S9** Swollen resistance of the 3D-printed hydrogel with MOFs nanoparticle self-reinforcing network. (**a**) Stress-strain and (**b**) load-unloading curves before and after one week of DI water swelling


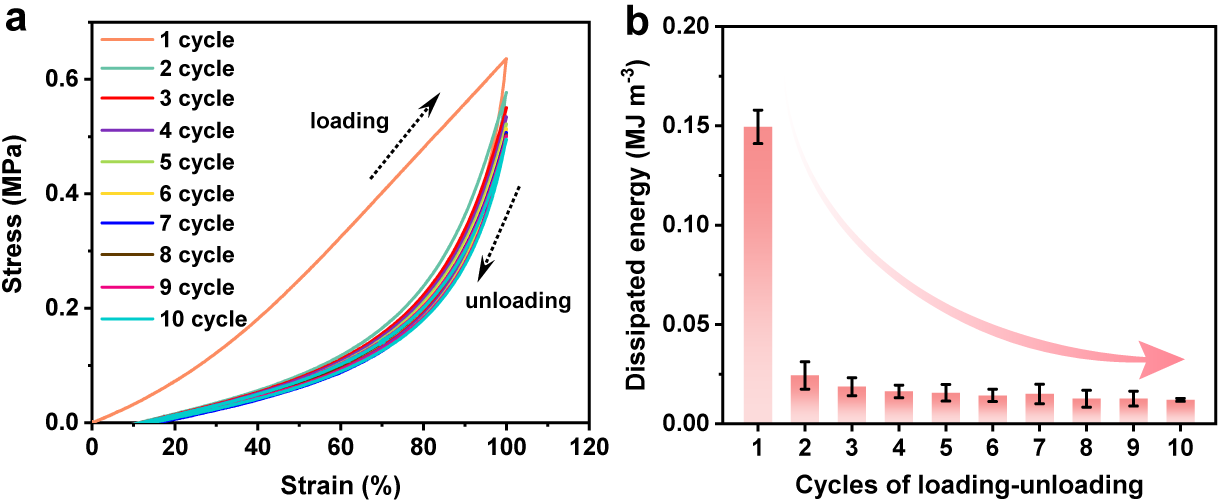


**Fig. S10** (**a**) Fatigue resistance performance of MOFs nanoparticles network hydrogel. (**b**) The corresponding energy dissipated under each cycle


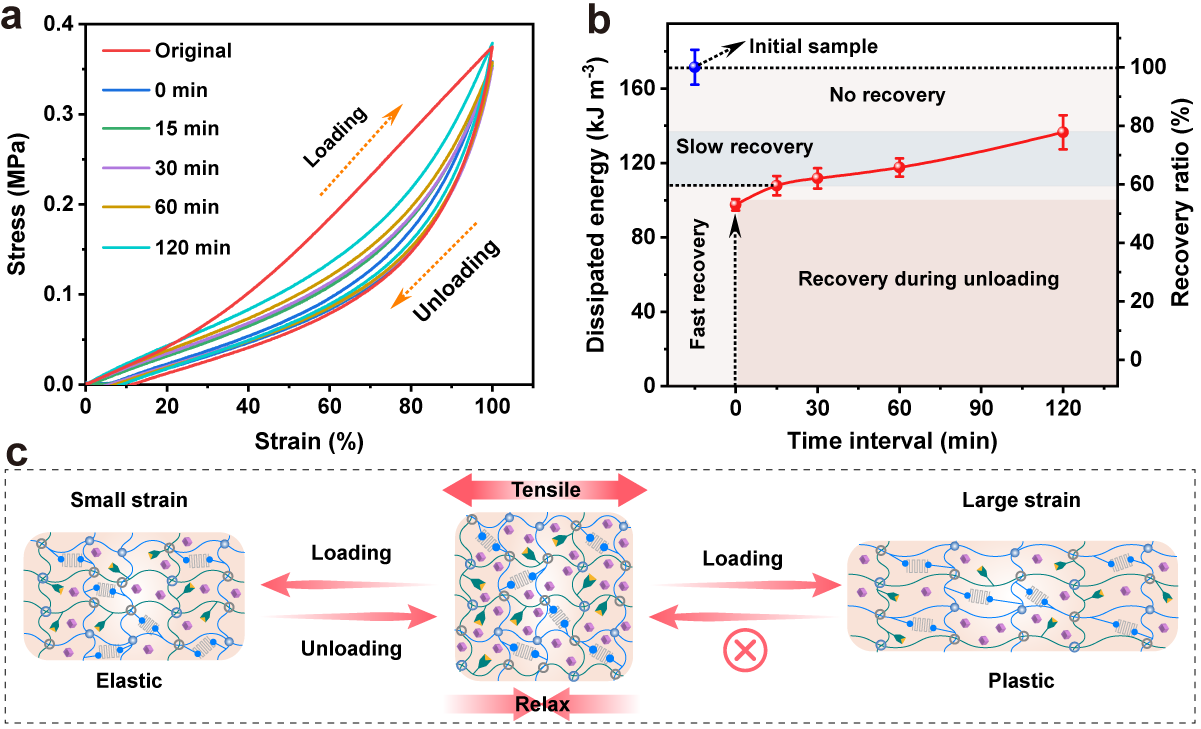


**Fig. S11** (**a**) Elastic recovery of MOFs nanoparticle network hydrogel under different waiting times. (**b**) Waiting time dependent-dissipated energy and recovery ratio. (**c**) Mechanism of elastic and plastic deformation of MOFs nanoparticle network hydrogels


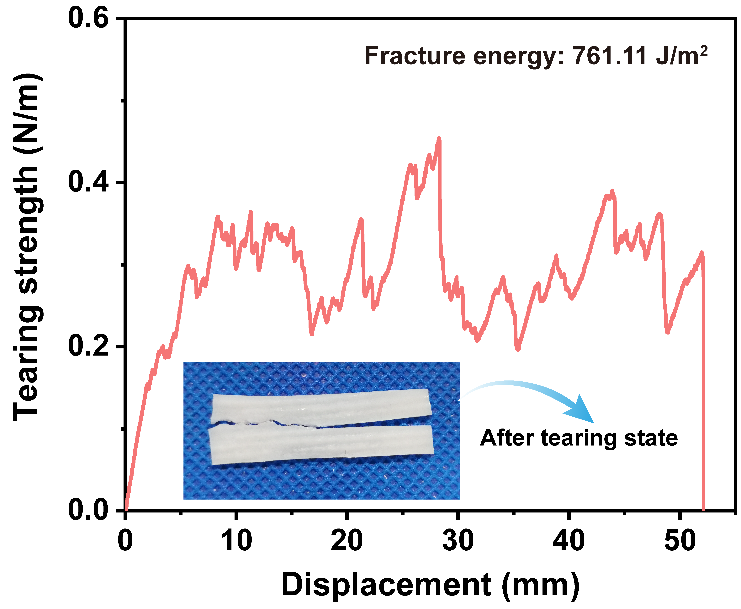


**Fig. S12** Tearing strength-displacement curves of hydrogels with MOFs nanoparticle self-reinforcing network and the corresponding fracture energy


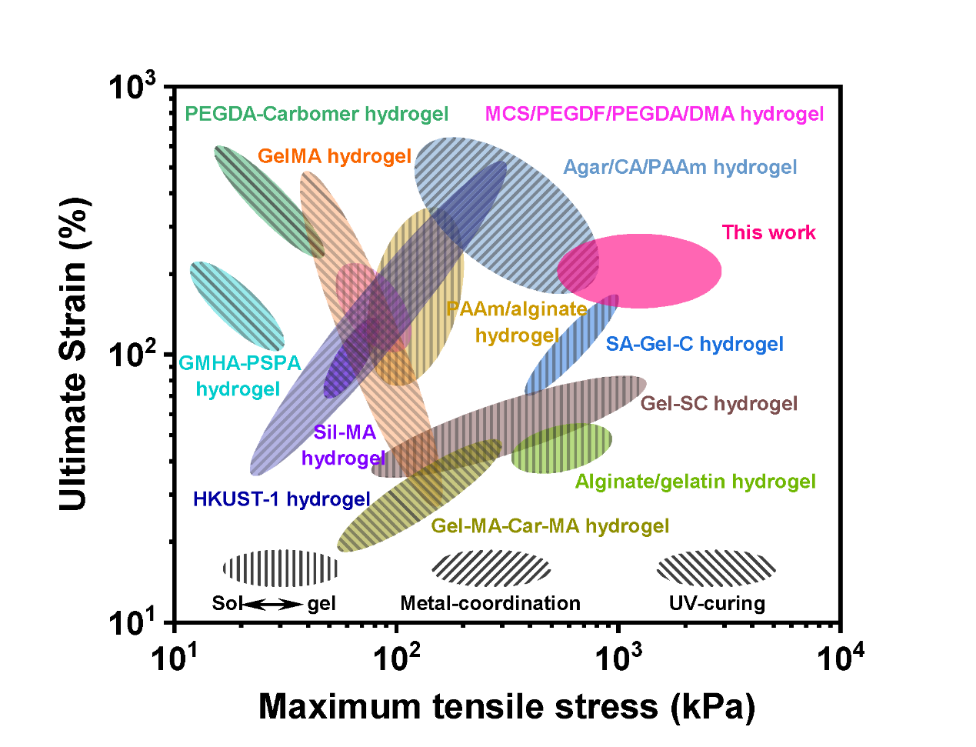


**Fig. S13** Comparison of fracture strain and tensile strength of MOFs nanoparticle network hydrogels and other ones

**Table S1** Comparison of fracture strain and tensile strength

| **Hydrogel samples** | **Fracture strength (kPa)** | **Ultimate strain (%)** | **Refs.** |
| --- | --- | --- | --- |
| *Gel-MA-Car-MA* | 60 | 20 | [S1] |
|  | 130 | 35 |  |
|  | 250 | 41 |  |
| *PAAm-Carbomer* | 17 | 547 | [S2] |
|  | 40 | 260 |  |
| *Sil-MA* | 52 | 77.6 | [S3] |
|  | 75 | 124.2 |  |
| *GelMA* | 40 | 420 | [S4] |
|  | 60 | 190 |  |
|  | 90 | 140 |  |
|  | 110 | 80 |  |
|  | 140 | 30 |  |
| *PEG-DA* | 10 | 160 | [S5] |
|  | 20 | 50 |  |
|  | 33 | 200 |  |
|  | 35 | 94 |  |
| *GMHA–PSPA* | 13.5 | 200 | [S6] |
|  | 15 | 180 |  |
|  | 18.5 | 155 |  |
|  | 20 | 130 |  |
|  | 27.5 | 125 |  |
| *HKUST-1* | 25 | 40 | [S7] |
|  | 30 | 50 |  |
|  | 135 | 300 |  |
|  | 277.6 | 453 |  |
| *Agar/CA/PAAm* | 385.56 | 223.63 | [S8] |
|  | 488.75 | 220.3 |  |
|  | 744.57 | 235.78 |  |
|  | 283.33 | 215.44 |  |
|  | 596.84 | 218.54 |  |
|  | 142.67 | 566.38 |  |
| *SA-Gel-C* | 900 | 150 | [S9] |
|  | 430 | 80 |  |
| *Gel-SC* | 90 | 38 | [S10] |
|  | 160 | 42 |  |
|  | 270 | 47 |  |
|  | 340 | 48 |  |
|  | 610 | 66 |  |
|  | 680 | 67 |  |
|  | 1100 | 75 |  |
| *PAAm/alginate* | 91 | 210 | [S11] |
|  | 170 | 300 |  |
|  | 110 | 150 |  |
|  | 130 | 90 |  |
|  | 140 | 220 |  |
| *Alginate/gelatin* | 840 | 50 | [S12] |
|  | 640 | 50 |  |
|  | 400 | 40 |  |
| *ZIF-8 nanoparticle network hydrogel* | 902.37 | 214.44 | This work |
|  | 2224.17 | 209.79 |  |
|  | 2501.4 | 199.02 |  |
|  | 2021.35 | 246.99 |  |
|  | 1257.16 | 162.92 |  |
|  | 577.14 | 205.79 |  |
|  | 1676.06 | 219.83 |  |
|  | 1716.83 | 229.82 |  |
|  | 2021.35 | 246.98 |  |
|  | 1841.1 | 204.97 |  |

**Table S2** Comparison of Young's modulus and tensile strength.

| **Hydrogel samples** | **Young’s modulus (kPa)** | **Fracture strength (kPa)** | **Refs.** |
| --- | --- | --- | --- |
| *Gel-MA – Car-MA* | 2.5 | 60 | [S1] |
|  | 2.4 | 130 |  |
|  | 2.3 | 250 |  |
| *Sil-MA* | 9.7 | 52 | [S3] |
|  | 14.5 | 75 |  |
| *GelMA* | 50 | 40 | [S4] |
|  | 75 | 60 |  |
|  | 100 | 90 |  |
|  | 125 | 110 |  |
|  | 150 | 140 |  |
| *PEG-DA* | 5.3 | 10 | [S5] |
|  | 74.6 | 20 |  |
|  | 12.7 | 33 |  |
|  | 42.5 | 35 |  |
| *GMHA–PSPA* | 30 | 13.5 | [S6] |
|  | 35 | 15 |  |
|  | 58 | 18.5 |  |
|  | 90 | 20 |  |
|  | 140 | 27.5 |  |
| *HKUST-1* | 65 | 25 | [S7] |
|  | 72 | 30 |  |
|  | 130 | 135 |  |
|  | 152.3 | 277.6 |  |
| *Agar/CA/PAAm* | 26.74 | 385.56 | [S8] |
|  | 38.14 | 488.75 |  |
|  | 55.45 | 744.57 |  |
|  | 17.14 | 283.33 |  |
|  | 41.2 | 596.84 |  |
|  | 16.29 | 142.67 |  |
| *SA-Gel-C* | 900 | 900 | [S9] |
|  | 500 | 430 |  |
| *Gel-SC* | 350 | 90 | [S10] |
|  | 540 | 160 |  |
|  | 850 | 270 |  |
|  | 1200 | 340 |  |
|  | 1900 | 610 |  |
|  | 2100 | 680 |  |
|  | 2900 | 1100 |  |
| *κ*-*carrageenan/PAAm DN* | 350 | 700 | [S13] |
|  | 275 | 600 |  |
|  | 200 | 600 |  |
|  | 120 | 550 |  |
|  | 75 | 400 |  |
|  | 50 | 210 |  |
| *Agar/PAAm DN* | 440 | 581.87 | [S14] |
|  | 550 | 662.5 |  |
|  | 810 | 781.25 |  |
|  | 870 | 1096.2 |  |
| *PAAm/alginate* | 48 | 91 | [S11] |
|  | 66 | 170 |  |
|  | 78 | 110 |  |
|  | 61 | 130 |  |
|  | 83 | 140 |  |
| *Alginate/gelatin* | 1140 | 840 | [S12] |
|  | 990 | 640 |  |
|  | 960 | 400 |  |
| *AMPS/AAm* | 69 | 160 | [S15] |
|  | 167 | 437 |  |
|  | 1016 | 1417 |  |
| *ZIF-8 nanoparticle network hydrogel* | 309.8 | 902.37 | This work |
|  | 586.8 | 2224.17 |  |
|  | 1012 | 2501.4 |  |
|  | 619.6 | 2021.35 |  |
|  | 570 | 1257.16 |  |
|  | 246.8 | 577.14 |  |
|  | 531.2 | 1676.06 |  |
|  | 505 | 1716.83 |  |
|  | 619.6 | 2021.35 |  |
|  | 778.2 | 1841.1 |  |

**Table S3** Comparison of Young's modulus and toughness

| **Hydrogel samples** | **Young’s modulus (kPa)** | **Toughness (kJ m^-3^)** | **Refs** |
| --- | --- | --- | --- |
| *HKUST-1* | 65 | 30 | [S7] |
|  | 72 | 40 |  |
|  | 130 | 300 |  |
|  | 152.3 | 744.7 |  |
| *Agar/CA/PAAm* | 26.74 | 479.71 | [S8] |
|  | 38.14 | 603.22 |  |
|  | 55.45 | 1049.77 |  |
|  | 17.14 | 362.01 |  |
|  | 41.2 | 762.02 |  |
|  | 16.29 | 493.27 |  |
| *Gel-SC* | 350 | 19 | [S10] |
|  | 540 | 35 |  |
|  | 850 | 73 |  |
|  | 1200 | 110 |  |
|  | 1900 | 140 |  |
|  | 2100 | 250 |  |
|  | 2900 | 660 |  |
| *Agar/PAAm DN* | 440 | 3.66 | [S14] |
|  | 550 | 0.95 |  |
|  | 810 | 3.86 |  |
|  | 870 | 1.61 |  |
| *PAAm/alginate* | 48 | 95 | [S11] |
|  | 66 | 260 |  |
|  | 78 | 83 |  |
|  | 61 | 59 |  |
|  | 83 | 154 |  |
| *AMPS/AAm* | 69 | 401.4 | [S15] |
|  | 167 | 302.4 |  |
|  | 1016 | 1060 |  |
| *ZIF-8 nanoparticle network hydrogel* | 309.8 | 1033.58 | This work |
|  | 586.8 | 2152.41 |  |
|  | 1012 | 2453.83 |  |
|  | 619.6 | 2415.14 |  |
|  | 570 | 967.07 |  |
|  | 246.8 | 606.43 |  |
|  | 531.2 | 1750.57 |  |
|  | 505 | 1888.25 |  |
|  | 619.6 | 2415.14 |  |
|  | 778.2 | 1863.01 |  |

**Table S4** Quantitative comparison with the existing state-of-the-art in lubricating hydrogels and meniscus substitute materials

| **Hydrogel samples** | **Fracture strength**  **(MPa)** | **Toughness**  **(MJ m^-3^)** | **Young’s modulus**  **(MPa)** | **Refs.** |
| --- | --- | --- | --- | --- |
| GMP-PNASC | 2.22~3.09 | / | 84.86~128.03 | [S16] |
| PNAGA | 0.91~2.13 | / | 0.15~0.30 | [S17] |
| PNASC-PCBAA | 0.35~4.34 | 1.01~25.49 | 2.03~10.92 | [S18] |
| NASC/Gln | 1.54~2.69 | / | 18~41 | [S19] |
| PVA/CMC/ZIF-8 | 1.68~2.50 | 1.75~2.45 | 0.59~1.10 | This work |

**Supplementary References**

1. L. Tytgat, L. Van Damme, M. del Pilar Ortega Arevalo, H. Declercq, H. Thienpont et al., Extrusion-based 3D printing of photo-crosslinkable gelatin and κ-carrageenan hydrogel blends for adipose tissue regeneration. Int. J. Biol. Macromol. **140**, 929–938 (2019). <https://doi.org/10.1016/j.ijbiomac.2019.08.124>
2. Z. Chen, D. Zhao, B. Liu, G. Nian, X. Li et al., 3D printing of multifunctional hydrogels. Adv. Funct. Mater. **29**(20), 1900971 (2019). <https://doi.org/10.1002/adfm.201900971>
3. S.H. Kim, Y.K. Yeon, J.M. Lee, J.R. Chao, Y.J. Lee et al., Precisely printable and biocompatible silk fibroin bioink for digital light processing 3D printing. Nat. Commun. **9**(1), 1620 (2018). <https://doi.org/10.1038/s41467-018-03759-y>
4. Y. Sun, K. Yu, J. Nie, M. Sun, J. Fu et al., Modeling the printability of photocuring and strength adjustable hydrogel bioink during projection-based 3D bioprinting. Biofabrication **13**(3), 035032 (2021). <https://doi.org/10.1088/1758-5090/aba413>
5. L.A. Hockaday, K.H. Kang, N.W. Colangelo, P.Y.C. Cheung, B. Duan et al., Rapid 3D printing of anatomically accurate and mechanically heterogeneous aortic valve hydrogel scaffolds. Biofabrication **4**(3), 035005 (2012). <https://doi.org/10.1088/1758-5082/4/3/035005>
6. Y.-W. Kang, J. Woo, H.-R. Lee, J.-Y. Sun, A mechanically enhanced electroactive hydrogel for 3D printing using a multileg long chain crosslinker. Smart Mater. Struct. **28**(9), 095016 (2019). <https://doi.org/10.1088/1361-665X/ab325d>
7. W. Liu, O. Erol, D.H. Gracias, 3D printing of an *in situ* grown MOF hydrogel with tunable mechanical properties. ACS Appl. Mater. Interfaces **12**(29), 33267–33275 (2020). <https://doi.org/10.1021/acsami.0c08880>
8. J. Wang, Y. Liu, S. Su, J. Wei, S.E. Rahman et al., Ultrasensitive wearable strain sensors of 3D printing tough and conductive hydrogels. Polymers **11**(11), 1873 (2019). <https://doi.org/10.3390/polym11111873>
9. L. Li, S. Qin, J. Peng, A. Chen, Y. Nie et al., Engineering gelatin-based alginate/carbon nanotubes blend bioink for direct 3D printing of vessel constructs. Int. J. Biol. Macromol. **145**, 262–271 (2020). <https://doi.org/10.1016/j.ijbiomac.2019.12.174>
10. S.E. Bakarich, R. Gorkin, R. Gately, S. Naficy, M. in het Panhuis et al., 3D printing of tough hydrogel composites with spatially varying materials properties. Addit. Manuf. **14**, 24–30 (2017). <https://doi.org/10.1016/j.addma.2016.12.003>
11. S.E. Bakarich, M.I.H. Panhuis, S. Beirne, G.G. Wallace, G.M. Spinks, Extrusion printing of ionic–covalent entanglement hydrogels with high toughness. J. Mater. Chem. B **1**(38), 4939–4946 (2013). <https://doi.org/10.1039/c3tb21159b>
12. B. Duan, L.A. Hockaday, K.H. Kang, J.T. Butcher, 3D Bioprinting of heterogeneous aortic valve conduits with alginate/gelatin hydrogels. J. Biomed. Mater. Res. Part A **101A**(5), 1255–1264 (2013). <https://doi.org/10.1002/jbm.a.34420>
13. S. Liu, L. Li, Ultrastretchable and self-healing double-network hydrogel for 3D printing and strain sensor. ACS Appl. Mater. Interfaces **9**(31), 26429–26437 (2017). <https://doi.org/10.1021/acsami.7b07445>
14. J. Wei, J. Wang, S. Su, S. Wang, J. Qiu et al., 3D printing of an extremely tough hydrogel. RSC Adv. **5**(99), 81324–81329 (2015). <https://doi.org/10.1039/C5RA16362E>
15. F. Yang, V. Tadepalli, B.J. Wiley, 3D printing of a double network hydrogel with a compression strength and elastic modulus greater than those of cartilage. ACS Biomater. Sci. Eng. **3**(5), 863–869 (2017). <https://doi.org/10.1021/acsbiomaterials.7b00094>
16. Z. Xu, Q. Zhang, C. Fan, M. Xiao, R. Yang et al., A gel microparticle-based self-thickening strategy for 3D printing high-modulus hydrogels skeleton cushioned with PNAGA hydrogel mimicking anisotropic mechanics of *Meniscus*. Bioact. Mater. **26**, 64–76 (2023). <https://doi.org/10.1016/j.bioactmat.2023.02.020>
17. 17 Z. Xu, C. Fan, Q. Zhang, Y. Liu, C. Cui et al., A self-thickening and self-strengthening strategy for 3D printing high-strength and antiswelling supramolecular polymer hydrogels as *Meniscus* substitutes. Adv. Funct. Mater. **31**(18), 2100462 (2021). <https://doi.org/10.1002/adfm.202100462>
18. C. Fan, Z. Xu, T. Wu, C. Cui, Y. Liu et al., 3D printing of lubricative stiff supramolecular polymer hydrogels for *Meniscus* replacement. Biomater. Sci. **9**(15), 5116–5126 (2021). <https://doi.org/10.1039/D1BM00836F>
19. R. Yang, C. Fan, Y. Dou, X. Zhang, Z. Xu et al., 3D printing stiff antibacterial hydrogels for *Meniscus* replacement. Appl. Mater. Today **24**, 101089 (2021). <https://doi.org/10.1016/j.apmt.2021.101089>
